# Exploring the accuracy of palaeobiological modelling procedures in forward-dynamics simulations of maximum-effort vertical jumping

**DOI:** 10.1098/rsos.242109

**Published:** 2025-05-21

**Authors:** Samuel R. R. Cross, James P. Charles, William I. Sellers, Jonathan R. Codd, Karl T. Bates

**Affiliations:** ^1^ Institute of Life course and Medical Sciences, University of Liverpool, Liverpool, UK; ^2^ School of Earth and Environmental Sciences, University of Manchester, Manchester, UK; ^3^ Faculty of Biology, Medicine & Health, University of Manchester, Manchester, UK

**Keywords:** jumping, simulation, multibody dynamics, model validation, human, bird

## Abstract

The body fossil record cannot preserve the dynamics of animal locomotion, and the only way to systematically reconstruct it is through simulation. However, musculoskeletal models used in simulation studies are typically simplified, meaning that their efficacy must first be demonstrated on living animals. Here, we evaluate a workflow for forward-dynamics simulations of maximum-effort vertical jumping, using simplified human and guineafowl models built with muscle masses from either measured data or estimated with methods previously applied to fossils. Predicted human performance was approximately 10% below experimental averages when known muscle masses were used, while the error ranged between +3 and −10% with palaeobiological methods. The simulations also correctly replicated the kinematic strategies (countermovement or squat jump) used across different starting postures. In contrast, predicted guineafowl performance was around 50–60% experimental values, irrespective of reconstruction method. Guineafowl model underperformance likely reflects simplifications related to foot mobility, muscle activation speeds and muscle fibre lengths, with the latter potentially being adaptively important to exceptional avian jumping performance. These findings emphasize that current muscle reconstruction and simulation approaches are most suited for evolutionary analyses where broad changes in body morphology and posture may significantly impact vertical jumping through pronounced qualitative differences in kinematic strategy.

## 1. Introduction

The mechanical performance of a biological structure, system or entire organism can be challenging to measure *in vivo*, and it is often necessary to estimate or predict performance metrics through biomechanical modelling (e.g. [1–7]). In the last two to three decades, computational advances have enabled the development of three-dimensional modelling techniques that attempt to replicate realistic mechanical and physiological environments *in silico*, thereby allowing for non-invasive quantification of many aspects of biomechanical performance (e.g. [8–10]). One such approach is multibody dynamic analysis (MDA), which is frequently employed in the study of robotic and biological motion, and (in biomechanical studies) typically integrates a linked (i.e. jointed) series of rigid body segments with muscular actuators that approximate the contractile behaviour of real skeletal muscle [11–17]. This musculoskeletal model is then combined with motion data, which may be derived either inversely from experimental forces or kinematics (e.g. [14,16]), or estimated via predictive simulations that optimize towards a particular target criterion (e.g. [11,12,17]). The universal relevance of this approach to studying the vertebrate musculoskeletal system has led to its application to a wide range of taxa and body plans, such as birds [14,18,19], non-avian dinosaurs [7,20–27] and mammals [17,28,29] including hominins [11,30–34]. In addition, biomechanical modelling, particularly in conjunction with predictive simulations, provides the sole avenue for dynamic gait reconstruction in extinct animals and thus offers insights into their locomotor faculty unafforded by more traditional comparative methods [4,7,22–26,31–36].

A practical necessity of MDA, like any modelling exercise, is that the final model will represent a simplification of reality, both in terms of its representation of the musculoskeletal system of a given taxon and its predicted biomechanical behaviour or performance. While this is partly an issue of computational demand, in the specific case of fossil animals, this additionally reflects the considerable uncertainties surrounding soft-tissue data [4,23,24,35,37,38]. Therefore, when modelling extinct taxa, researchers often estimate or generalize aspects of the muscular system, such as muscle volumes and masses [4,7,20–22,24,34–36], fibre and tendon lengths [4,21–24,35,36,39] and the actual pathways of the muscles themselves, which are often condensed into a suite of functional actuators regulating limb motion(s) along a given axis [4,20–22,31,35]. It follows then, that model validation must guide the assessment of this approach, by demonstrating that these models can maintain their quantitative efficacy in the analytical context in which they are to be used, despite considerable departure from ‘real’ animal anatomy and physiology.

Previous work has shown that muscle mass is one of the most important parameters to estimate accurately when modelling fossil animals [4,20–24,35,37,38], because both the power and force (via the relationship with physiological cross-sectional area (PCSA)) available to a muscle, are proportional to its mass [40,41]. Numerous methods have been developed to estimate muscle masses (or PCSA) either schematically [3,4,7,20–22,35], predictively [34,42,43], via geometric scaling [30,44] or through manual volumetric sculpture [45–48]. These often yield qualitatively and quantitatively divergent values, even within a single method if it allows for the introduction of subjective error [39]. Estimating muscle mass has obvious implications for model performance and therefore functional inference [23,37,38]; however, many simulation-based studies of fossil taxa have tended to utilize mass value(s) derived from a single method only [7,20–22,24,27,30,31], and even when more than one approach has been used to estimate muscle mass or PCSA, it remains unknown how close subsequent predictions of locomotor performance are to the real values for extinct animals. Such an assessment of predictive accuracy can only be achieved by applying reconstruction methods to living animals where biomechanical performance has been measured.

Vertical jumping in humans has been extensively analysed with optimal control simulations, which have provided new insights into numerous biomechanical aspects, including neuromuscular control [12,49–51], elastic energy storage and utilization [52–54] and force development [54–57]. Owing to its clear target criterion (‘raise your centre of mass as high as possible’) and relatively low computational demands compared with steady-state locomotion, jumping represents an attractive subject for dynamic optimization in animals (including fossils), despite receiving limited attention to date [18]. Jumping is an important component of the locomotor repertoire of many taxa and often plays a key role in predator evasion, prey capture and other activities that require rapid bursts of movement [58–62]. Previous work has shown that limb posture can directly influence the kinematic strategy and performance of vertical jumping, particularly in terms of height and speed [51,54,57,63–68], which would be expected to have direct ecological consequences and thus selective potential in regular jumpers (e.g. birds and primates). Given the considerable interest in the macroevolution of posture and locomotor mode (again, birds [69–71] and primates [34,72,73] being obvious examples), investigation of a co-evolutionary relationship between posture and jumping abilities using forward dynamics simulations has clear merit. However, because jumping is often a maximum-effort activity, where muscles are expected to be performing at or near their peak [74], it is crucial that the impact of model simplification and muscle mass estimation is evaluated before any large-scale evolutionary analyses are undertaken.

Here, we examine the predictive accuracy of simulations of maximum vertical jumping in two extant bipedal animals (humans and guineafowl) using models that have been constructed with methods typically employed in fossil-based evolutionary studies. Simulated performance data (including jump heights, impulses and kinematics) was compared against published performance data (for the guineafowl) and new subject-specific experimental performance data (for the human). These experimental data on jumping performance allowed us to assess the absolute quantitative accuracy of simulations in both models. In addition, we also simulated jumping from different starting postures with the human model, since this is known to yield differences in many aspects of jumping performance. By comparing these postural simulations with our experimental data, we examined the model’s capacity to accurately predict relative or qualitative differences between postural conditions, which has relevance to palaeontological studies seeking to test macroevolutionary hypotheses related to postural evolution in fossil lineages.

## 2. Material and methods

### 2.1. Subject-specific *in vivo* human jumping

An adult male (*n* = 1; mass = 68.4 kg) was fitted with 52 reflective markers (following a similar layout to [75]) and filmed jumping barefoot from three separate starting postures on a force plate (Kistler 9281E), using a 12-camera Qualisys Oqus 7 motion capture system (Qualisys Inc., Götenborg, Sweden). Trials were conducted in sets of 10, with the subject rested for 1 min between individual jumps. The three starting postures (and respective mean starting joint angles) were full extension (Hip 10°; Knee 0°; Ankle −5°), a shallow crouch (Hip 67°; Knee −58°; Ankle 6°) and deep crouch (Hip 114°; Knee −95°; Ankle 11°), of which 10 individual jumps were performed for each posture. During a trial, the subject moved onto the force plate, performed the jump at a self-selected interval and then moved away to rest. The subject was instructed to attain the maximum possible vertical height during each jump, without further direction regarding particular jumping strategies (e.g. squat versus countermovement mechanics [54]), besides the requirement to keep their hands by their side to eliminate the performance increase from arm swing [76]. The motion capture data were then labelled in Qualisys (v. 2.15.) and exported to OpenSim (v. 4.3.) to derive joint kinematics via inverse kinematic analysis of a subject-specific model of the same adult male (this model was first introduced by Charles *et al*. [15]). All experiments were approved by the University of Liverpool Research Ethics Committee (Reference Number: RETH001005).

Joint kinematic and force plate data were exported to R (v. 4.2.2. [77]) for processing, using a combination of force and positional criteria to identify the major phases of each jump. The start of a jump was defined as a change in force by 5 and −5% body weight for squat and countermovement mechanics respectively, while the end of a jump was considered the instance where the maximum vertical position of the centre of mass (CoM) was achieved. The decision to omit the declining and landing phases of the jump was based upon our simulation protocol, which optimizes for maximum height and not landing performance. We defined take-off as the instance where the vertical forces dropped to 0 (indicating the subject was aerial), and for countermovement jumps the transition between eccentric (downwards) and concentric (upwards) phases, as an increase in CoM height following its lowest position.

We evaluated several parameters commonly used as performance metrics in studies of human jumping [54,67,78,79]; the maximum vertical position of the CoM (i.e. jump height), which represents the vertical distance travelled by the CoM relative to the floor; the concentric displacement distance, which is the total vertical distance travelled by the CoM between its lowest position and take-off, and corresponds to the distance (and therefore time) available for limb extension and impulse generation [63,65–68,79,80]; the relative net vGRF impulse (RNVI), which was calculated for the concentric phase only, and taken as the impulse arising from the vertical ground reaction forces divided by body mass [67,68,78,79]; the vertical velocity of the CoM at take-off (i.e. take-off velocity); the time taken between the start of the jump sequence and take-off (i.e. contact time); and lastly, sagittal plane angular kinematics of the hip, knee and ankle.

### 2.2. Simulations of bipedal jumping

#### 2.2.1. Model assembly

**Figure 1.**
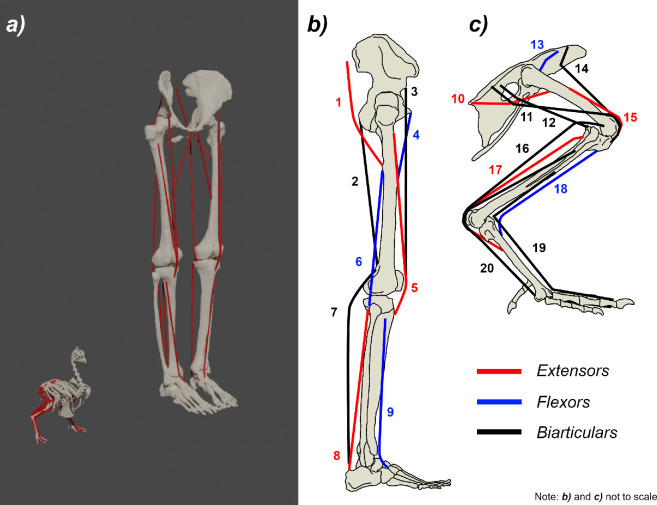
Human and guineafowl musculoskeletal models. (*a*) Final in-scale models of each taxon illustrating three-dimensional skeletal and reconstructed muscular anatomy. (*b*) Diagram of human actuator (aggregated muscle) pathways, identity and approximate guide muscle as follows; 1, Hip extensor (M. gluteus maximus); 2, Hip extensor knee flexor (M. semitendinosus); 3, Hip flexor knee extensor (M. rectus femoris); 4, Hip flexor (M. adductor longus); 5, Knee extensor (M. vastus intermedius); 6, Knee flexor (M. biceps femoris); 7, Knee flexor ankle plantarflexor (M. gastrocnemius medialis); 8, Ankle plantarflexor (M. soleus); 9, Ankle dorsiflexor (M. tibialis anterior). (*c*) Diagram of guineafowl actuator (aggregated muscle) pathways, identity and approximate guide muscle as follows; 10, Hip extensor (M. caudofemoralis pars pelvica); 11, Hip extensor knee extensor (M. iliotibialis lateralis pars postacetabularis); 12, Hip extensor knee flexor (M. iliofibularis); 13, Hip flexor (M. iliotrochantericus cranialis); 14, Hip flexor knee extensor (M. Iliotibialis cranialis); 15, Knee extensor (M. femorotibialis medialis); 16, Knee flexor ankle extensor (M. gastrocnemius lateralis); 17, Ankle extensor (M. gastrocnemius medialis); 18, Ankle flexor (M. tibialis cranialis); 19, Ankle flexor pes dorsiflexor (M. extensor digitorum longus); 20, Ankle extensor pes plantarflexor (M. flexor digitorum longus). Note that for *b*) and *c*) many actuators share similar points of insertion. All illustrations by the authors.

Models were constructed of two living bipeds, a human and a helmeted guineafowl (*Numida meleagris*, hereafter ‘guineafowl’), according to the established protocol for GaitSym 2017 (figure 1; [7]). The human was first introduced as a GaitSym model by Bates *et al*. [34] and is derived from an MRI scan of the same individual used in the experimental study, with a final model mass of 68.33 kg (figure 1*a*). The guineafowl model was built for this analysis and originates from a CT scan by Macaulay *et al*. [71], which was repositioned for simulation (sigmoidal neck, tucked wings and 10° hip adduction) and has a final model mass of 1.38 kg (figure 1*a*). Initial skeletal realignments were performed in Blender (v. 2.90.1. [81]), with mass and inertial properties calculated for each body segment in GaitSym 2017. Segmental densities were assumed to be 1000 kg m^−3^, with the exception of the head and torso (850 kg m^−3^), and neck (800 kg m^−3^), which were lowered to account for the presence of airways [71].

Hindlimb joint centres were fitted via manual approximation in Blender (v. 2.90.1. [81]), guided by previous human and avian reconstructions [15,18–20,34], and ensuring a permissible range of motion minimally equivalent to the experimental trials while avoiding serious intersegmental collisions [7,22,46]. Joints were then modelled as fixed hinges permitting flexion-extension only. While in both humans and birds jumping is three-dimensional, previous simulations of jumping have tended to restrict movement to these axes [49,51,52,55,57,65], since fewer operational axes reduce the search space but can still yield realistic results in high-performance activities (e.g. [20,34]). The hip, knee and ankle joints were included for both taxa, with the addition of a tarsometotarsophalangeal joint (TMTP) for the guineafowl. In both models, the functional foot (i.e. the human pes and avian phalanges), the forelimb, and the axial skeleton were kept rigid. Joint limits are presented in electronic supplementary material, S1. For the human model, we used the existing limits of Bates *et al*. [34], but these were estimated for the guineafowl based on the range of motion (RoM) presented in Henry *et al*. [82]. This approach has several caveats. Firstly, Henry *et al*. [82] calculated the hip angle relative to external dorsal markers, which required us to estimate the starting position of this joint as horizontal (i.e. the femur is parallel to the pelvis). Secondly, preliminary testing of these ranges found that several extreme values were likely unfeasible for our model and resulted in considerable inter-segmental collision, so we tuned these by eye to more appropriate ranges. Note, however, that all joints were modelled with soft limits, meaning that they could marginally exceed their ranges while a damped constraining force is applied.

Following Sellers *et al*. [7], we included limb bone stress constraints in both models. The method is described in full by the original authors, but briefly this approach calculates the linear forces and rotational torques acting about a fixed midshaft ‘joint’, from which bone stress is calculated following Alexander [83]. This method requires knowledge of the midshaft cross-sectional profile of the long bones, which was taken from our original three-dimensional scan data. In the human model, both femoral and tibial-fibular stress were calculated, whereas femoral, tibiotarsal and tarsometatarsal stresses were calculated for the guineafowl. Midshaft stress analysis helps constrain potential kinetic and kinematic strategies, since solutions that exceed the long bone safety factors involve skeletal failure and are therefore discarded [7].

Contacts were placed on the distal limb of both models to represent interactions between the model and the floor. These use standard parameter values (see Sellers *et al*. [7,32]), and behaved like stiff, damped springs that allowed the foot to be lifted without resistance. In total, 18 contacts were placed beneath each foot of the human model, while 10 were placed beneath the pes in the guineafowl, and another postero-distally on the tarsometatarsus.

Muscles were modelled following the same multibody dynamic approach used in previous studies [7,20–23,27,31,32,34], using a Hill-type muscle model with linear serial and parallel elastic elements [84]. Limb muscles were not modelled individually, rather the entire musculature was aggregated into a series of functional actuators that represent the pooled effect of all muscles performing a given action about a given joint (figure 1). To reduce intersections with the skeletal geometries and other actuators, the line-of-action of each actuator was guided by either via points or wrapping cylinders where appropriate and usually followed the approximate origin, insertion and pathway of a major (usually largest) muscle of the corresponding functional group (see figure 1*b,c*). In total, 9 aggregated muscles were modelled for the human model and 11 for the guineafowl model. Muscle fibre and tendon lengths were calculated from the length change of each actuator across its working range [21]. PCSA was calculated by dividing the muscle volume by the estimated fibre length [22,40,41] (the process of muscle volume/mass estimation is detailed in the next section). Maximum shortening velocity (*V*_max_) was kept at 8.4 L s^−1^ for the human model (as set by Bates *et al*. [34]) but was increased to 14 L s^−1^ for the guineafowl based on Nelson *et al*. [85]. Limb objects (joints, contacts and muscles) were symmetrized prior to simulation.

#### 2.2.2. Model variants and fossil estimation methods

For each taxon, we produced three model variants representing the differing levels of uncertainty in soft-tissue estimation when working with extant versus fossil animals. Given our specific interest in the influence of muscle mass estimation, we kept all actuator properties constant besides PCSA to isolate the effects of different reconstruction methods. The muscle masses assigned to each actuator were measured/estimated according to the variant-specific criteria outlined below and converted to a volume assuming a muscle density of 1056 kg m^−3^ for calculation of PCSA [40]. A summary overview of each model is provided in table 1, while final actuator properties for each taxon and model combination are outlined in table 2.

**Table 1.** Summary of main methodological differences between the models *1−3* of each taxon, including the guineafowl *Model 1* sensitivity analyses (identified as '*M1 –'*). For a kinematic breakdown of the different starting postures (i.e. initial joint angles) see electronic supplementary material, table S1, and for numerical differences in muscle PCSA arising from the differing measurements/estimations refer to main text table 2. The protocol for estimating actuator fibre and tendon lengths follows the range of motion approach of [21] for all main analytical models (i.e. models *1−3*) though we employed additional methods in our sensitivity analysis of avian jumping.

model	starting posture(s)	muscle mass data	fibre protocol	tendon protocol
human				
*model 1*	full extension (habitual) shallow crouch deep crouch	measured—subject-specific	RoM method [21]	RoM method [21]
*model 2*	full extension (habitual) shallow crouch deep crouch	estimated—schematic bipedal intermediate	RoM method [21]	RoM method [21]
*model 3*	full extension (habitual) shallow crouch seep crouch	estimated—predicted from muscle attachment areas	RoM method [21]	RoM method [21]
guineafowl				
*model 1*	crouched (habitual)	measured—taxon-specific (scaled)	RoM method [21]	RoM method [21]
*M1* - deeper crouch	hypothetical deeper crouch	measured—taxon-specific (scaled)	RoM method [21]	RoM method [21]
*M1* - optimized tendons	crouched (habitual)	measured—taxon-specific (scaled)	RoM method [21]	uniarticular extensors allowed to vary by up to +/−10%, elsewise RoM method [21]
*M1* - FL / 2	crouched (habitual)	measured—taxon-specific (scaled)	RoM method divided by 2	RoM method [21]
*M1* - FL × 2	crouched (habitual)	measured—taxon-specific (scaled)	RoM method multiplied by 2 [21,22]	RoM method [21,22]
*M1* - FL × 2 + optimized tendons	crouched (habitual)	measured—taxon-specific (scaled)	RoM method multiplied by 2 [21,22]	uniarticular extensors allowed to vary by up to +/ −10%, elsewise RoM method [21,22]
*model 2*	crouched (habitual)	estimated—schematic bipedal intermediate	RoM method [21]	RoM method [21]
*model 3*	crouched (habitual)	estimated—predicted from muscle attachment areas	RoM method [21]	RoM method [21]

**Table 2.** Estimated properties for the functional actuators present in each model/taxon. Fibre lengths estimated via the range of motion method [21] are presented under ‘**FL**’ and given in mm. The model-specific PCSAs for each actuator, which were calculated for each model according from its estimated muscle masses, are presented in **M1** (*Model 1*), **M2** (*Model 2*) and **M3** (*Model 3*), and are given in mm^2^. The parentheses in **M2** and **M3** indicate the percentage difference between the corresponding value of **M1**. All values are presented for a single limb. Abbreviations for our actuator naming scheme are, ‘ext’ = extensor; ‘flx’ = flexor; ‘pfx’ = plantarflexor, and ‘dfx’ = dorsiflexor.

taxon	actuator	FL	M1	M2	M3
human	hip ext	172.295	5534.583	8125.023 (147%)	6195.388 (112%)
	hip ext knee flx	123.632	6444.721	14425.111 (224%)	3052.271 (47%)
	hip flx	45.389	33435.136	16607.251 (50%)	19982.571 (60%)
	hip flx knee ext	138.213	1913.731	13183.589 (689%)	880.254 (46%)
	knee ext	129.079	13922.225	8276.688 (59%)	15344.503 (110%)
	knee flx	108.875	1454.071	3522.453 (242%)	1022.853 (70%)
	knee flx ankle ext	59.142	7891.753	20811.168 (264%)	19720.55 (250%)
	ankle ext	50.426	16678.017	16803.977 (101%)	35014.739 (210%)
	ankle flx	28.742	6597.919	31747.279 (481%)	38448.22 (583%)
	*total*	*NA*	*93872.155*	*133501.539 (142%)*	*139661.349 (149%)*
guineafowl	hip ext	20.854	566.144	930.188(164%)	282.115 (50%)
	hip ext knee ext	33.814	594.958	1003.959 (169%)	116.584 (20%)
	hip ext knee flx	29.682	1457.053	1049.824 (72%)	289.241 (20%)
	hip flx	8.426	1625.218	1861.331 (115%)	559.835 (34%)
	hip flx knee ext	42.968	343.063	703.599 (205%)	109.607 (32%)
	knee ext	15.383	1326.183	945.784 (71%)	272.122 (21%)
	knee flx ankle ext	18.790	948.424	1401.101 (148%)	362.748 (38%)
	ankle ext	17.639	992.099	825.64 (83%)	635.110 (64%)
	ankle flx	18.304	280.401	856.851 (306%)	215.809 (77%)
	pes dfx	18.263	18.289	429.374 (2348%)	156.694 (857%)
	pes pfx	24.436	186.948	595.992 (319%)	522.668 (280%)
	*total*	*NA*	*8338.781*	*10603.64 (127%)*	*3522.532 (42%)*

*Model 1*. This model uses measured (real) muscle mass data from the studied taxa. For the human model, we used our subject-specific MRI muscle data originating from previous work [15], and also used in Bates *et al*. [34]. Subject-specific data were not available for the guineafowl; however, several earlier studies have documented hindlimb muscle masses for this taxon [82,86,87], so we used the muscle data of Cox *et al*. [87], which yields the highest ratio of limb muscle mass to total body mass and scaled their data isometrically to our model. Individual muscle masses were assigned to actuators representing their specific function in the flexion-extension plane (see table 2), using an identical scheme to Bates *et al*. [34] for the human, and van Bijlert *et al*. [19] for the guineafowl.

*Model 2*. This model represents an attempt to replicate the fairly generalized, schematic approach to muscle estimation that is regularly employed in biomechanical modelling [3,4,7,20–22] and is primarily concerned with functional/mechanical considerations as opposed to factors such as phylogenetic relatedness. This specific model follows the approach of Sellers *et al*. [20–22] for partitioning muscle mass across the limb joints, in combination with measured muscle data for extant striding bipeds (birds and humans) from Hutchinson [3]. Total limb muscle mass was considered to be 35% of total body mass, based on Hutchinson’s [3] finding that the hindlimbs of striding bipeds comprise approximately 17% total body mass per limb on average. This muscle mass is then allocated to each joint following a proximal-distal partitioning of 40% hip, 30% knee, 20% ankle and 10% TMTP, which derives from the bird–human average of Hutchinson’s [3] data (note that if the overall striding biped average was used instead, the higher number of birds versus the single human would distort this partitioning scheme towards a distally enlarged, very avian condition). The amount of muscle assigned to extension (including plantarflexion) and flexion at each joint is divided using a 65:35 ratio, again following Hutchinson’s [3] finding that approximately 67% of limb mass in striding bipeds comprises extensors muscles (and assuming the remaining 33% will primarily comprise the flexors). The muscle mass per joint is then divided equally between the actuators that act across it for a given action, as per previous studies [21,22].

*Model 3*. Previous work [34,43] has shown that with varying degrees of confidence, it is possible to predict muscle properties (masses, PCSA) in extinct taxa from corresponding bone surface areas (either attachment site areas or gross bone areas (e.g. shaft area)). For the human model, we used the bone area predictive equations originally devised by Bates *et al*. [34] for estimating hindlimb muscle masses in fossil hominins. Briefly, this uses generalized bone areas (approximately corresponding to attachment areas and muscle pathways) to predict muscle masses through linear regression of these parameters in extant hominoids. This approach has one notable caveat, in that a human average was included within the analysis of Bates *et al*. [34], so our estimations are partly derived from conspecific data. However, given the marked differences between *Model 1* and *Model 3* (table 2), we consider this a minor consideration that does not particularly undermine any of the objectives of this study.

A similar approach was taken for the guineafowl *Model 3*, but using the archosaur equations from Cuff *et al*. [43], which differ from those of Bates *et al*. in several fundamental ways. Instead of using generalized bone areas, Cuff *et al*. use the actual attachment areas of muscles (the origins, specifically) to predict PCSA directly (not mass). Defining attachment areas is (as detailed by Cuff *et al*. [43]) fairly challenging, and therefore a combination of muscle-specific equations (10 total) and a common equation for the remaining muscles were developed by the original authors. For the guineafowl, muscle origin areas were subjectively identified and measured in Blender (v. 2.90.1. [81]) and subsequently log-transformed and entered into the relevant predictive equation to estimate the corresponding PCSA. There are several points of note with this approach relative to our other models. For one, since PCSA is estimated directly here, it is not calculated with our estimated muscle fibre lengths, which is the case elsewhere. In addition, Cuff *et al*. [43] do not analyse the intrinsic muscles of the pes, meaning that their data do not incorporate several of the digital dorsiflexors, which are included in our model. We have therefore utilized their common scaling equation to provide a tentative estimate for this group; however, given that the digital dorsiflexors are both very small and not involved in limb extension (which is much more critical to jump performance), we do not consider this a significant limitation.

#### 2.2.3. Choice of model starting postures

The human model was positioned into three distinct starting postures using joint angles that approximate the starting postures used in the experimental trials (these were determined by preliminary experimental trials and are within 10° of the mean final experimental starting angles at each joint) (table 1; electronic supplementary material, S1). For the guineafowl, a single starting posture approximating the initial joint angles presented in Henry *et al*. [82] was used in the main analysis, while a more flexed posture was evaluated as a sensitivity analysis (with the caveat that no experimental data exists against which to evaluate its predicted performance) (table 1; electronic supplementary material, S1).

#### 2.2.4. Sensitivity analysis of avian jumping

Our primary simulations of guineafowl jumping were found to substantially underperform relative to the experimental data, which likely reflects a combination of diverging kinematic strategies and misrepresentation of muscle–tendon properties (this is described in full in the discussion). Therefore, a series of guineafowl *Model 1* variants were developed and simulated to address each of these concepts and attempt to determine their relative importance (a summary overview of each sensitivity variant is also provided in table 1).

The first variant retained the original muscle properties of *Model 1* but was placed into a more deeply flexed starting posture using joint angles that approximate the deepest level of overall crouch (the end of the countermovement) used by the birds experimentally [82] (table 1). Since the original *Model 1* squat jumps without a countermovement, it omits several centimetres of potential concentric displacement distance, which could result in a reduction in jump height.

Muscle fibre lengths have a large impact upon force generation and are therefore an important model attribute for sensitivity analysis. This study originally calculated fibre length as the change in muscle length experienced across the maximum permissible range of joint motion in the model [21], and this makes an assumption that the fibre is relatively well tuned to generate a reasonably large amount of force across its entire working length (or in other words, it is operating on the optimal portions of its force–length curve across the entire anatomically possible range of joint motion), which is unlikely to be realistic for most muscles [16,22,88]. Previous work [22] has shown that a tuning factor of 2, which doubles fibre length, is a reasonable approximation for many vertebrate skeletal muscles. We implemented this approach as a second model variant, multiplying fibre lengths by 2 and recalculating PCSAs and tendon lengths (table 1). However, there is some evidence to suggest that avian muscles operate over narrower ranges than in humans [19,89], which would align with their capacity to generate high forces very rapidly [82,90,91] and might enhance vertical jumping from their fairly crouched postures. Therefore, for a third model variant the fibre lengths of the distal limb muscles were instead tuned by a factor of 0.5 (table 1). During initial testing, we extended this tuning to the proximal limb muscles as well; however, this led to high femoral stresses that exceeded the 300 MPa limit and caused the simulation to fail.

Tendons are thought to play an important role in enhancing jumping performance through their capacity for elastic storage and release [18,52,82,92,93]. Here, tendon length is calculated relative to initial fibre length [7,21,22], and it is possible that this will lead to over- or underestimations of tendon length, which may allow for unrealistic elastic returns or impact upon the contractile ability of the muscular component. While the fibre length sensitivity models described earlier will investigate tendon changes to some extent (shortening or lengthening fibres would, respectively, increase or decrease tendon length in our workflow), it may also be insightful to also consider this attribute in isolation. Therefore, for a fourth model variant we included tendon length within the optimization parameters, allowing the tendons of the uniarticular extensor muscles to vary by +/−10% of their original estimated length, which represents modest limits that should be sufficient to explore the potential issues further and identify strategies for improvement (table 1). In other words, in these simulations the model is free to select tendon lengths of the uniarticular extensor muscles that are anywhere within the range of +/−10% of their original values, which lead to greater jump heights. Finally for a fifth model variant, we combined this tendon optimization approach with the longer muscle fibres (i.e. tuned by a factor of 2), which serves to investigate the interplay between these two features (table 1).

#### 2.2.5. Simulation procedure

Simulations were performed in GaitSym 2017 [7], using a genetic optimization procedure to find patterns of muscle activations that maximize vertical height within a given time limit. In contrast to previous steady-state analyses (e.g. [7,21,22,34]), jumping consists of a singular movement, so left and right muscle activations were symmetrized and kept in-phase. During the early testing phase of this study, we found that overall jump performance was strongly influenced by our choice of time limit. For example, if the simulation was allowed to run for a longer duration than needed to perform a jump, the algorithm would regularly generate patterns that ‘filled up’ this excess time, often leading to a reduction in performance. We therefore chose to include a sensitivity analysis of time limit to ensure reasonable heights and kinematics were being sampled and tested four separate taxon-specific time limits (see electronic supplementary material, S2). In total, 25 independent simulations were run for each combination of taxon, muscle estimation method, starting posture and time limit, amounting to more than 1200 simulations overall.

#### 2.2.6. Comparisons of simulated and experimental jumps

Every simulated jump was checked manually (visually) in GaitSym, and performance data were exported for analysis in R (v. 4.2.2. [77]). Since the core theme of this study is an assessment of model accuracy, we calculated the identical parameters to those outlined earlier for the experimental study, which permits direct comparisons with both our subject-specific human experimental data and that of Henry *et al*. [82] for the guineafowl. The influence of time limit was evaluated first, by comparing the best simulations for each property combination (taxon, starting posture), to identify the optimal time limit needed to produce maximal jump height. Following this, the most optimal solution for *Models 1−3* was compared directly against the experimental data.

It is important to note that the guineafowl jump heights presented in Henry *et al*. [82] are not maximum CoM heights (i.e. true jump heights) but rather the height of the perch the birds’ jumped to, including occasional supplementary flapping. Since our goal is to simulate leg-driven jumping (our model cannot flap), this could make comparisons with our results challenging. However, it is possible to predict final CoM height from vertical take-off velocity using the following equation [94]


$$CoM_{\text{max}}=CoM_{\text{take-off}}+\left(\frac{V_{\text{take-off}}^{2}}{2g}\right)$$

where *CoM*_max_ is the maximum height achieved by the CoM (i.e. jump height), *CoM*_take-off_ is the height of the CoM at take-off, *V*_take-off_ is the vertical velocity of the CoM at take-off and *g* is the gravitational acceleration. Using the values for these parameters reported by Henry *et al*. [82] (CoM_take-off_ = 0.25 m; *V*_take-off_ = 3.3 m s^−1^), and assuming a *g* of 9.81 m s^−2^, the predicted jump height is 0.81 m, which is marginally lower than the perch height at 0.84 m.

## 3. Results

### 3.1. Human jumping

A preliminary sensitivity analysis of simulation time limit was undertaken prior to the main analysis (electronic supplementary material, S2). Four separate time intervals were analysed for each posture, using *Model 1* muscle properties, and these showed either a plateau or decline in maximum jump height by the highest interval, which allowed us to identify the best time limit(s) for simulating each model and posture (electronic supplementary material, figure S2a).

**Figure 2.**
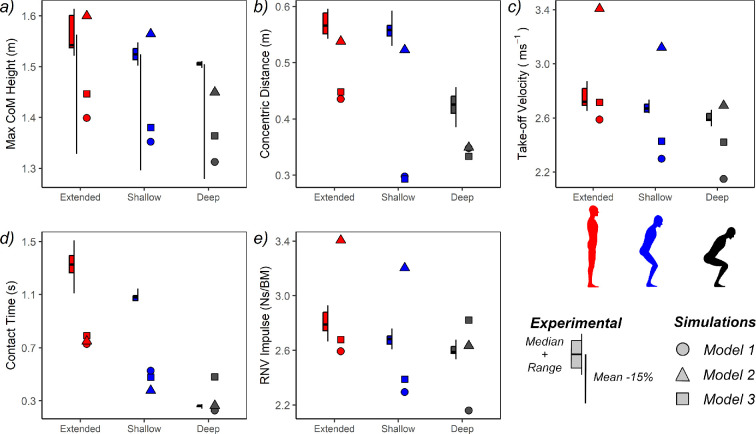
Discrete performance characteristics of subject-specific human jumping from three starting postures. Experimental data are presented as boxplots to the left of each posture (median + range), and for jump height *a*), an additional line has been included for comparative purposes, which represents the −15% bounds on the experimental average. The result for the most optimal simulation for *Models 1−3* is presented to the right of each posture. All illustrations by the authors.

With *Model 1,* we sought to quantify the impact of the GaitSym modelling procedure and general simplifications to model anatomy (e.g. aggregated muscles and rigid functional foot segment) while retaining reasonably realistic (subject-specific) muscle properties. Comparisons of extensor PCSA showed that *Model 1* had a similar total PCSA to our subject, albeit with approximately 20% less acting in extension overall, and this being fairly distally skewed in terms of its distribution across the joints (table 3). Our simulation results also showed that the most optimal solutions for this model underperformed relative to their experimental equivalent across every starting posture (figure 2; table 4), though maximum CoM height remains comfortably within 15% of the experimental mean (extended −10%, shallow crouch −11% and deep crouch −13%; figure 2*a*; table 4), and maxima (−13% all starting conditions; figure 2*a*; table 4) and within 10% of several experimental minima (extended −8%, shallow crouch −10% and deep crouch −12%; figure 2*a*; table 4). While reduced absolute or quantitative performance was ubiquitous across the parameters investigated, the relative or qualitative ordering of performance across the starting postures is usually upheld between the experiments and simulations. The sole exception to this was concentric displacement distance (figure 2*b*; table 4). In both experiments and simulations, maximum jump height (figure 2*a*; table 4), take-off velocity (figure 2*c*; table 4), contact time (figure 2*d*; table 4) and relative net vertical impulse (figure 2*e*; table 4) were all found to be greatest when starting from full extension, followed by the shallow crouch and then the deep crouch (table 4). In contrast, a similar concentric displacement distance was found between extended and shallow crouched starts in the experiments, which was much higher than the deep crouch. This was not replicated by our simulations, which produced a disproportionately low concentric distance for the shallow crouch that overturned the qualitative order (figure 2*b*; table 4).

**Table 3.** Comparison of total PCSA (**PCSA^tot^**), total extensor PCSA (**Ext^tot^**) and joint extensor PCSA (**Joint^tot^**), for each taxon/model (in mm^2^). Alongside PCSA values for each of the three models developed here, we have also included real PCSA values (i.e. real muscle volumes/masses and fibre lengths) of both taxa for comparative purposes. For the human, this is derived from Charles *et al*. [15] and comprises the same subject used here, while for the guineafowl, the values are calculated from Cox *et al*. [87] for a 1.45 kg bird. In keeping with our models, we did not incorporate fibre pennation angles into PCSA calculations for either taxon, though do note that the fibre lengths provided in [87] are optimized, and therefore real guineafowl PCSA is likely to differ compared with the values presented here. For **Ext^tot^**, parentheses indicate proportion of **PCSA^tot^** composed of extensor PCSA, and for **Joint^tot^**, this instead represents the proportion of that joints’ extensor PCSA relative to **Ext^tot^**.

taxon	model	PCSA^tot^	Ext^tot^	Hip^tot^	Knee^tot^	Ankle^tot^	TMTP^tot^
human	Charles *et al*. [15]	88114.322	65620.846 (75%)	19964.169 (30%)	20414.646 (31%)	25242.031 (39%)	*NA*
	*model 1*	93872.155	52385.029 (56%)	11979.304 (23%)	15835.956 (30%)	24569.77 (47%)	*NA*
	*model 2*	133501.539	81624.555 (61%)	22550.134 (28%)	21460.277 (26%)	37614.145 (46%)	*NA*
	*model 3*	139661.348	80207.705 (57%)	9247.659 (12%)	16224.757 (20%)	54735.289 (68%)	*NA*
guineafowl	Cox *et al*. [87]	4496.616	3501.069 (78%)	1189.922 (34%)	1341.665 (38%)	1105.554 (32%)	184.634 (1%)
	*model 1*	8338.781	6414.872 (77%)	2618.155 (41%)	2264.204 (35%)	1940.523 (30%)	186.942 (3%)
	*model 2*	10603.64	7456.222 (70%)	2984.106 (40%)	2653.342 (36%)	2226.741 (30%)	595.922 (8%)
	*model 3*	3522.532	2706.799 (77%)	687.939 (25%)	498.313 (18%)	997.859 (37%)	522.668 (19%)

**Table 4.** Maximum-effort vertical jumping performance for the human experimental trials and simulations. Experimental results consist of the average of 10 individual trials per starting posture, whereas the model results represent the best simulation of that model/posture overall. Results comprise maximum CoM height (i.e. jump height) (m), concentric displacement distance (m), take-off velocity in (ms^−1^), contact time (s) and relative net vertical impulse (Ns per body mass). Kinematic strategy provides a general description of the kinematic approach used in each trial/simulation, 'CMJ' = countermovement jump, 'SJ' = squat jump.

model	max CoM height	concentric distance	take-off velocity	contact time	RNV impulse	kinematic strategy
*experiments*						
full extension	1.563	0.569	2.750	1.327	2.799	CMJ
shallow crouch	1.525	0.558	2.666	1.077	2.681	CMJ
deep crouch	1.505	0.424	2.602	0.260	2.599	SJ
*model 1*						
full extension	1.399	0.436	2.588	0.728	2.594	CMJ
shallow crouch	1.352	0.298	2.297	0.527	2.295	CMJ
deep crouch	1.313	0.348	2.149	0.227	2.161	SJ
*model 2*						
full extension	1.600	0.538	3.408	0.749	3.406	CMJ
shallow crouch	1.565	0.523	3.120	0.377	3.204	CMJ
deep crouch	1.450	0.349	2.691	0.263	2.635	SJ
*model 3*						
full extension	1.447	0.448	2.715	0.791	2.680	CMJ
shallow crouch	1.380	0.293	2.428	0.478	2.389	CMJ
deep crouch	1.364	0.333	2.421	0.480	2.821	SJ

*Models 2* and *3* replaced known muscle masses with those estimated according to methodologies designed (primarily) for fossils. When muscle masses were derived according to a schematic bird–human average, the total PCSA available to *Model 2* increased substantially (152% *Model 1* PCSA; table 2) and proportionally more PCSA was found to act in joint extension (with a distribution similar to the real subject; table 3). This led to a pronounced increase in jump performance over *Model 1* (extended +14%, shallow crouch +16% and deep crouch +11% relative to *Model 1*; figure 2*a*; table 4), with concentric displacement distance (figure 2*b*; table 4), take-off velocity (figure 2*c*; table 4) and relative net vertical impulse (figure 2*e*; table 4) all tending to exceed *Model 1* while maintaining an identical qualitative order. This comparatively high performance of *Model 2* included performance very close to the experimental averages (extended +2%, shallow crouch +3% and deep crouch −4%), and within the experimental range (figure 2*a*; table 4). Conversely, when muscle mass was predicted via a hominoid scaling equation, *Model 3* PCSA is similarly overestimated (159% *Model 1* PCSA; table 2), though strongly skewed about the ankle and both relatively and absolutely diminished at the proximal joints (table 3). This translates to only moderate increases in performance compared with *Model 1* (figure 2; table 4), with jump height only marginally greater in *Model 3* compared with *Model 1* (extended +3%, shallow crouch + 2% and deep crouch +4%; figure 2*a*; table 4), while concentric displacement difference was mostly equivalent (figure 2*b*; table 4). Similar results are found for the other parameters when jumping from extended or shallow crouched postures; however, the deep crouch was found to use a relatively long contact period (figure 2*d*; table 4), in association with a relatively high impulse (figure 2*e*; table 4) and take-off velocity (figure 2*c*; table 4), which overturns the qualitative ordering between it and the shallow crouch for several parameters (figure 2*b*,*d,e*; table 4). *Model 3* performance was not notably higher than *Model 1*, meaning this model also underperformed relative to the experiments (extended −7%, shallow crouch −10%, and deep crouch −9%; figure 2*a*; table 4).

**Figure 3.**
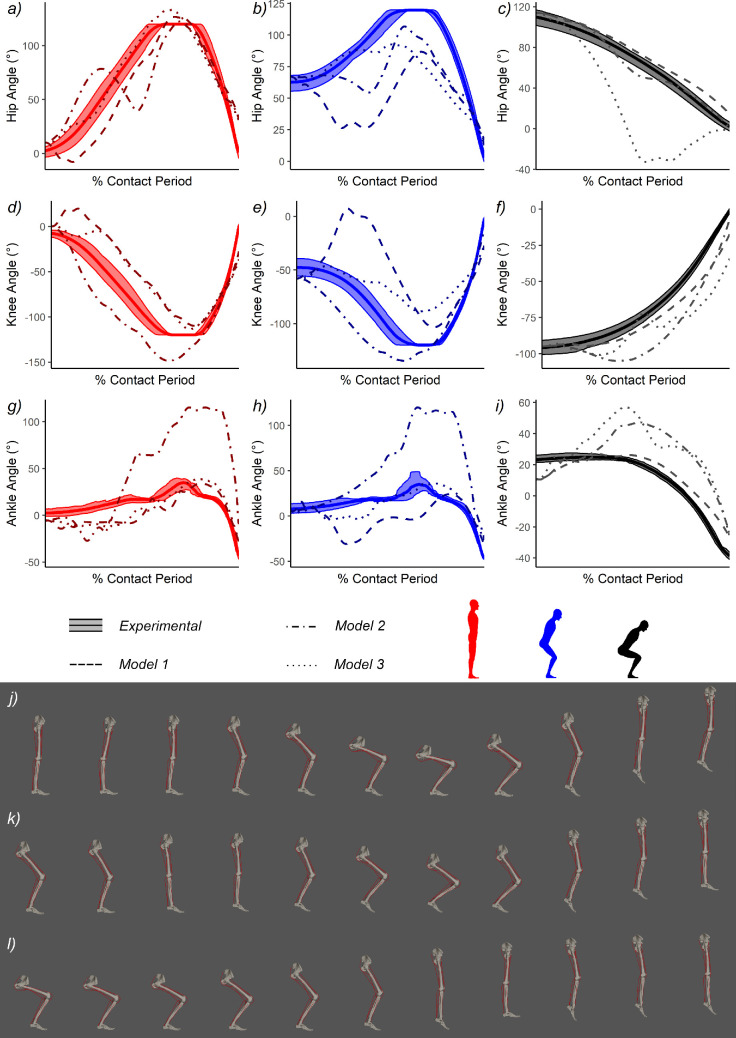
Joint kinematic traces of subject-specific human jumping from three starting postures (*a-i*). The experimental data are presented as a mean and range, whereas for the simulations (*Models 1−3*) only the most optimal solution is presented for each model. Rows correspond to specific joints, and from top to bottom comprise; hip (*a-c*), knee (*d-f*), and ankle (*g-i*). Columns correspond to starting postures and from left to right comprise; full extension *a,d,g*), a shallow crouch (*b,e,h*) and a deep crouch (*c,f,i*). For *Model 1* simulations, three-dimensional body kinematics of the most optimal simulation are presented in *j-l*) following full extension *j*) ,shallow crouch (*k*) and deep crouch (*l*). The image series is presented in relative time as 0–100% maximum jump height, increasing at 10% intervals. All illustrations by the authors.

Similar to the discrete parameters, contact-phase joint kinematics also showed pronounced differences between starting postures and modelling methods (figure 3; table 4). In the experimental trials, joint RoM was notably higher when jumping from full extension or a shallow crouch, since an episode of increasing flexion occurs at the start of the sequence. This indicates use of a countermovement, which was absent when jumping from a deep crouch (where squat jump mechanics are used instead; [54]) and is particularly notable at the hip (figure 3*a*–*c*), and knee (figure 3*d*–*f*), but less so at the ankle (figure 3*g*–*i*). The same general qualitative patterns were used by *Model 1*, as both extended and shallow crouched postures use a countermovement, whereas the deep crouch performs a squat jump. However, these countermovements are preceded by a period of initial hip and knee extension, and all starting postures are shown to take-off with more flexed limbs than found during the experiments (figure 3). *Model 2* continued to use a similar proximal kinematic strategy (without initial hip and knee extension prior to countermoving), though its ankle kinematics are characterized by extreme dorsiflexion that departs substantially from the experiments and other models (figure 2*g*–*i*). *Model 3* was found to produce arguably the most realistic countermovement kinematics when jumping from extended and shallow crouched postures. However, the deep crouch was shown to perform an unusual variation on a squat jump, where the hip is extended rapidly early in the take-off sequence and the ankle and knee flexed more slowly, leading to the knee still being significantly flexed at take-off (figure 2*f*).

### 3.2. Guineafowl jumping

As for the human model, a preliminary sensitivity analysis was first undertaken to identify the optimal time limits for simulations (electronic supplementary material, S2). A clear performance plateau was found between the two longest of the four tested time limits, indicating the best time limit(s) had been sampled (electronic supplementary material, figure S2b).

**Figure 4.**
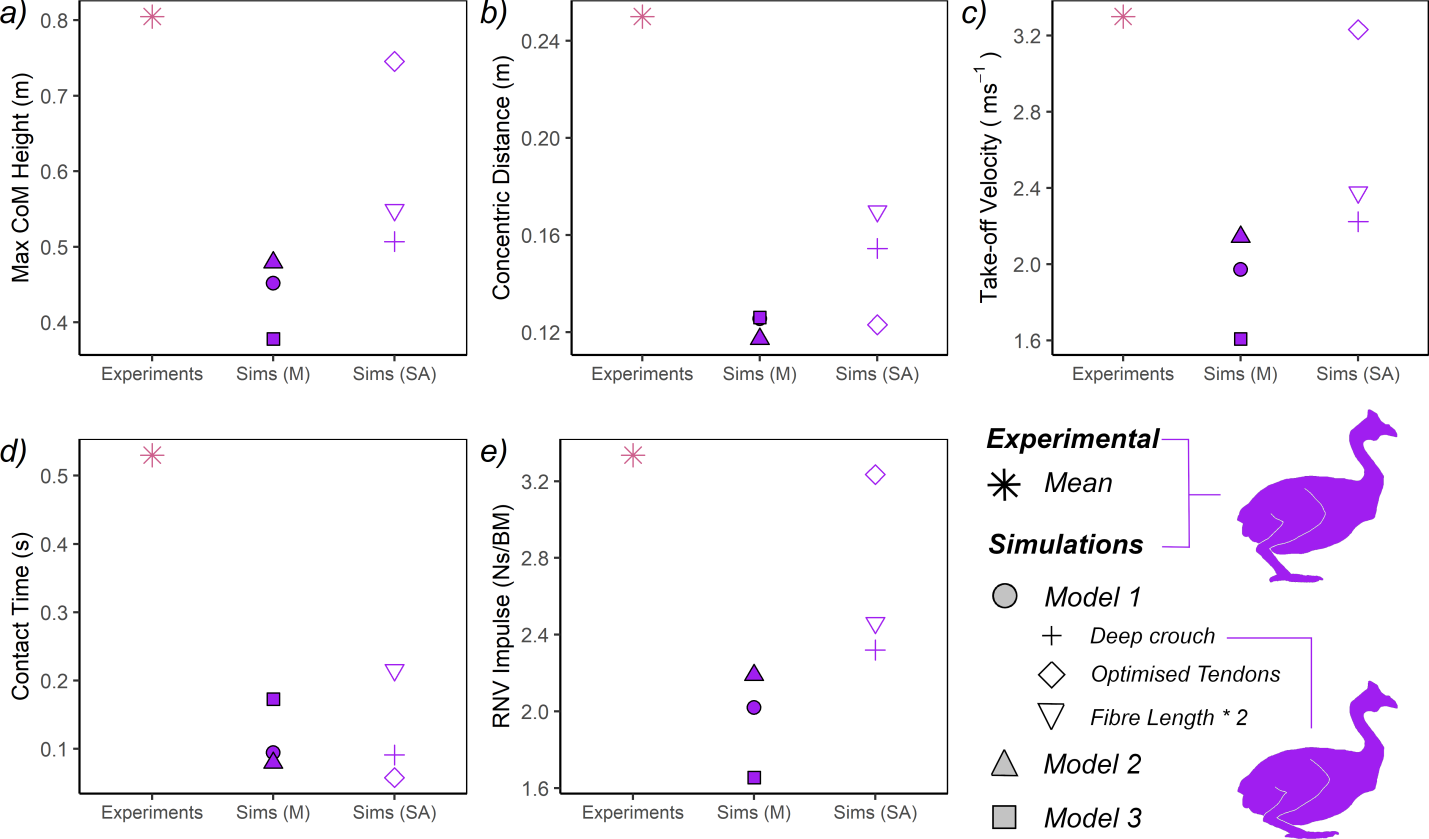
Discrete performance characteristics of simulated guineafowl jumping compared with the experimental data of Henry *et al*. [82]. Experimental data are presented as averages, while results of the simulations are split into two categories: main analysis (Sims M), which comprises the results of *Models 1−3* or sensitivity analyses (Sims SA), which include *Model 1* variants that address issues relating to posture and muscle physiology (see main text Material and methods and table 1). For all simulations, only the result of the most optimal solution is presented. All illustrations by the authors.

**Figure 5.**
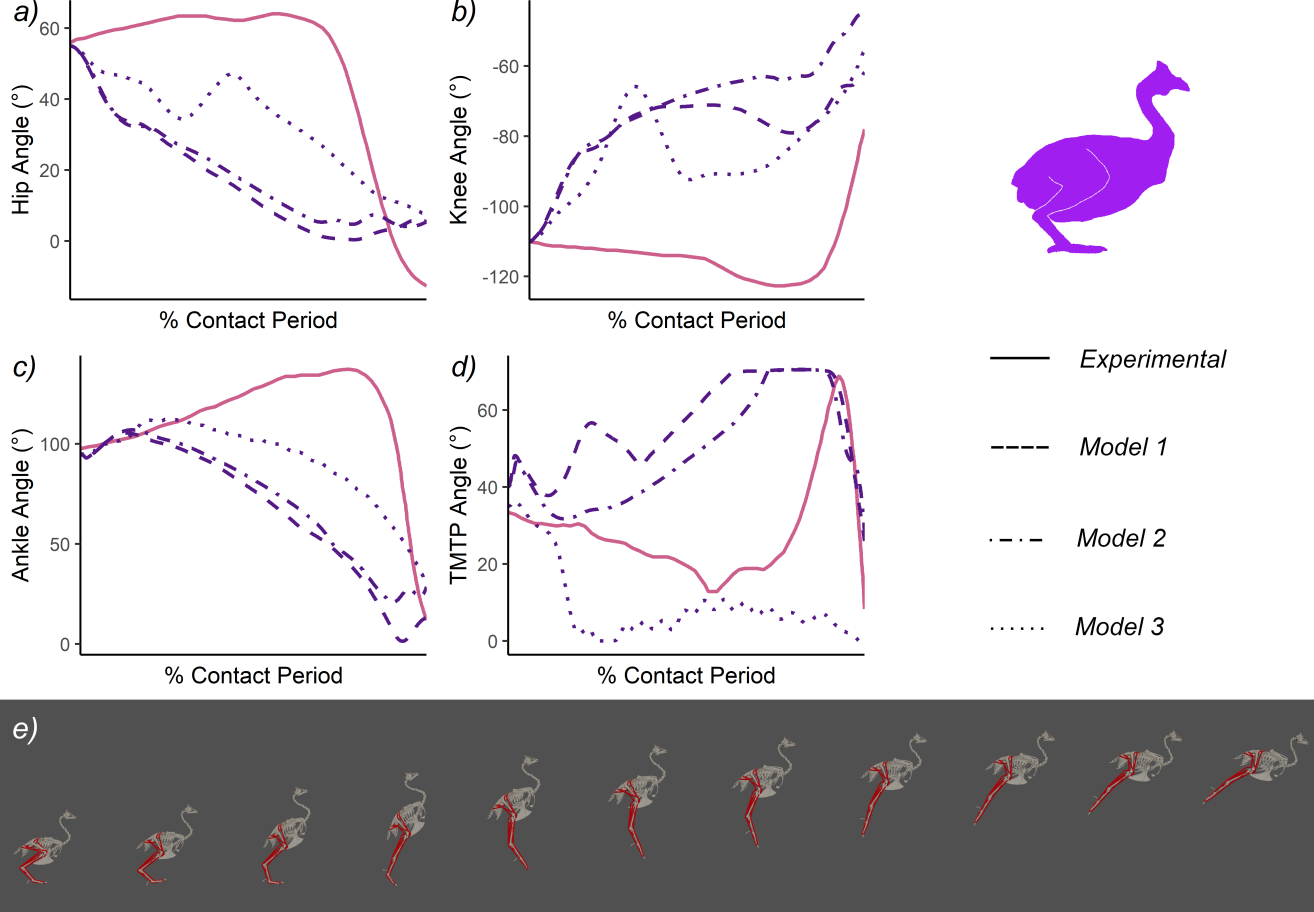
Joint kinematic traces of simulated guineafowl jumping (*Models 1−3*) compared with the experimental data of Henry *et al*. [82] *a-d*). For all simulations, only the result of the most optimal solution is presented. Three-dimensional body kinematics of *Model 1* is presented in *e*), these images document 0–100% maximum jump height at 10% intervals. All illustrations by the authors.

Compared with real guineafowl [87], *Model 1* had substantially more PCSA acting in limb extension, though proportionally this was concentrated more proximally about the hip (table 3). Surprisingly however, *Model 1* guineafowl performance was markedly low, with maximum jump height only 56% of the height predicted from Henry *et al*. [82] (figure 4*a*; table 5). This underperformance extends to the other parameters, which were all substantially lower than their experimental equivalents (figure 4*b*–*e*; table 5). In terms of kinematics, the *Model 1* guineafowl was shown to use a squat jump with continuous joint extension, which contrasts the countermovement strategy employed by the birds experimentally (figure 5; table 5) and is likely associated with the severely diminished concentric distance (figure 4*b*; table 5) and contact times (figure 4*d*; table 5).

**Table 5.** Maximum-effort vertical jumping performance for the guineafowl experimental trials (from [82]) and simulations. Model results represent the best simulation of that model/posture overall, whereas experimental data consist of averages across multiple individuals. For *Model 1*, the sensitivity analyses of this model are given as ‘*M1’* plus a short description (see table 1 for a more detailed breakdown). Presented results comprise maximum CoM height (i.e. jump height) (m), concentric displacement distance (m), take-off velocity in (ms^−1^), contact time (s) and relative net vertical impulse (Ns per body mass). Kinematic strategy provides a general description of the kinematic approach used in each trial/simulation, 'CMJ' = countermovement jump, 'SJ' = squat jump.

model	max CoM height	concentric distance	take-off velocity	contact time	RNV impulse	kinematic strategy
*experiments* [82]	0.810	0.250	3.300	0.530	3.336	CMJ
*model 1*	0.452	0.126	1.973	0.095	2.020	SJ
*M1* - deeper crouch	0.507	0.154	2.224	0.091	2.320	SJ
*M1* - optimized tendons	0.746	0.123	3.231	0.058	3.235	SJ
*M1* - FL / 2	0.441	0.118	1.955	0.089	2.099	SJ
*M1* - FL × 2	0.549	0.170	2.378	0.215	2.462	CMJ
*M1* - FL × 2 + optimized tendons	0.563	0.168	2.405	0.228	2.461	CMJ
*model 2*	0.479	0.117	2.145	0.080	2.190	SJ
*model 3*	0.378	0.126	1.608	0.173	1.655	SJ

Guineafowl *Model 2* achieved marginally higher jumps than *Model 1* (+6% *Model 1* jump height; figure 4*a*; table 5), while *Model 3* produced moderately lower jumps (−16% *Model 1* jump height; figure 4*a*; table 5). The extensor PCSA available to these models varied massively, being far greater (*Model 2*) and far lower (*Model 3*) than real guineafowl (table 2–3[87]), yet this did not have a large effect on absolute performance (there is only a 10 cm or 21% difference between the lowest (*Model 3*) and highest (*Model 2*); figure 4*a*; table 5). In addition, both models essentially follow the same squat jumping strategy employed by *Model 1* (figure 5; table 5), though *Model 3* did depart noticeably in terms of hip, knee and TMTP kinematics (figure 5*a,b,d*).

A sensitivity analysis of the parameters potentially contributing to this underperformance identified muscle fibre length and tendon length as two significant factors that (with sometimes only modest changes) could appreciably impact upon predicted jump height (figure 4*a*; table 5). Using longer fibre lengths (length change × 2; [7,22]) resulted in continued underperformance relative to the experiments (−32%), but a +21% increase in jump height relative to *Model 1* (figure 4*a*; table 5), alongside an incipient countermovement similar to the experimental data. In contrast, using shorter fibre lengths (and thus higher PCSAs) in the distal muscles (length change / 2) resulted in a minor (−2%) decrease in performance relative to the original *Model 1* and thus a similar decrease relative to the experiments (−45%; table 5). Allowing tendon lengths to be optimized within +/−10% of their original (i.e. *Model 1*) values led to a preferential shortening of all tendons (between −9.7 and −10%; electronic supplementary material, table S2) and a +65% performance increase over *Model 1*, which approaches the experimental jump heights at −7% (figure 4*a*; table 5). Interestingly, combining this tendon optimization criterion with the shorter muscle fibres led to much smaller gains in jump higher over *Model 1* (+25%), greater underperformance relative to the experiments (−30%) and only marginal improvements when compared with the original fibre length × 2 model (+3%) (table 5). Changes to tendon length in this model were also more variable, with the hip extensor being lengthened by + 5%, the pes plantarflexor being maximally shortened (−10%), while the knee and ankle extensors remained largely unchanged (electronic supplementary material, table S2). In addition, starting the model from a more deeply crouched posture (approximately equivalent to the countermovement depth used experimentally) increased jump height by approximately 6 cm, meaning this model still underperformed the experiments by −37% (figure 4*a*; table 5).

Further investigation of the impact of altering fibre and tendon properties across these models (electronic supplementary material, S3) showed that, on average, models with longer fibres tended to shorten at velocities closer to optimality (i.e. approx. 1/3 *V*_max_ [84]), and most positive work was done by the contractile element of their actuators (electronic supplementary material, table S3). In contrast, models with shorter fibres tended to shorten at velocities far in excess of theoretical optima, with a greater contribution from the series and parallel elastic elements to total work (electronic supplementary material, table S3). Of particular note was the tendon optimization model (with the original fibre lengths), which performed the most positive work overall (in keeping with its relatively high jump height; figure 4, table 5), despite incredibly rapid (and suboptimal) shortening of its contractile element. When considering the division of overall work done by each muscle–tendon component, it was apparent that this performance increase is related to absolute and relative increases in work done by the elastic elements (particularly the parallel element), whereas work done by the contractile element was reduced in both aspects (electronic supplementary material, table S3).

## 4. Discussion

Biomechanical modelling and simulation can be a powerful tool for studying form and function in the fossil record. However, despite the insights this approach can offer, the considerable uncertainties of modelling the musculoskeletal anatomy and physiology of an extinct taxon make methodological assessment of these approaches on living animals pertinent. Here, we investigated the quantitative and qualitative accuracy of multi-body dynamic simulations of maximum-effort vertical jumping in bipedal animals, using two living taxa (humans and guineafowl) that represent major lineages of obligate bipeds, whose fossil representatives have frequently been analysed using predictive simulations [7,20,23–26,30,31,34]. We demonstrated that a common modelling and simulation workflow, used in combination with known muscle masses [7,20–23,30–32,34], can reproduce many qualitative aspects of vertical jumping in both taxa; though absolute predicted performance varies considerably, being far closer to experimental values in the human (within −10 to −13% mean jump heights) as opposed to avian model (−44%). When muscle masses and PCSAs are replaced with values estimated according to fossil-based methods, quantitative jumping performance usually remains within 20% of the original simulations, even if the force-generating capacity of both models varies substantially. We suggest that timely coordination of muscle activations and joint rotations may enforce a degree of kinematic conformity across simulations irrespective of differences in PCSA. Further improvements to the accuracy of our guineafowl model can likely be made by addressing the relatively greater uncertainties and assumptions introduced during its construction. In particular, our results suggest that avian jumping is fairly sensitive to the choice of muscle fibre and tendon lengths, which appear to underscore birds' comparatively higher experimental performance than humans.

### 4.1. Accuracy of the human model with real muscle masses

The modelling process itself (e.g. rigid body segments) along with various practical choices made here (e.g. aggregated muscles) result in many simplifications or abstractions to musculoskeletal anatomy and mechanics, even with subject-specific input data. While many model attributes, such as skeletal proportions, mass properties and gross muscle pathways can either be directly measured or estimated with reasonable confidence from skeletal/fossil material (e.g. [71,95]), estimating intrinsic muscle properties (including muscle masses) is more challenging. Therefore, by utilizing known muscle masses, *Model 1* served to assess the impact of the overall modelling procedure, without some of the (potentially considerable) uncertainties associated with muscle estimation [3,4,15,22,24,34,35,41]. With this in mind, we found that our modelling workflow resulted in a approximately 20% reduction in extensor PCSA between *Model 1* and our experimental subject (table 3), suggesting slightly lower force-generating abilities in the model, which can account for much of its relatively low simulated jumping performance (figure 2; table 4). Maximum jump heights for *Model 1* are consistently within 15% of the experimental average across all three starting postures, with the extended start found to be the most accurate (−10%), followed by the shallow crouch (−11%) and then the deep crouch (−13%), which places two of the three postures (extended and shallow crouch) within 10% of their experimental ranges (figure 2*a*). The general tendency towards marginal underperformance was also found for the other parameters, and barring the sole exception of concentric distance, the same qualitative ordering of performance across the three postures is maintained (figure 2*b*–*e*; table 4). Previous work [34] has demonstrated that simulations of *Model 1* can reproduce maximum running speeds within the subject-specific experimental range, and these results indicate similar (albeit lower) performance at vertical jumping from multiple starting postures.

Limb posture is hugely important to locomotor function (e.g. [96]) and has been either a primary or ancillary focus of several earlier predictive simulation studies [19,20,31,33,97]. Vertical jumping is no exception, and starting posture is known to have a large impact on performance because it regulates the initial concentric distance and therefore time available for force generation, which directly influences the impulse and take-off velocity, and ultimately final jump height [57,63,65–68,80]. It follows then that jumping strategies may vary across species with different habitual standing or steady-state locomotor postures. At full extension, potential concentric muscle work is minimized and jump heights will be low. However, by incorporating a large countermovement, an animal starting from a more extended limb posture can increase their concentric distance and time available for force generation and thereby raise maximum height [18,54,55,63,66–68]. From an evolutionary perspective, it may then be hypothesized that postural changes could correlate significantly with changes to jumping strategy and/or performance, given this potential for stark differences in the overall strategic context (particularly the inherent trade-off between take-off time and overall jump height/distance [63–65,67]), which may be extremely important to the selective fitness of certain clades and taxa [58–62]. Indeed, many crouched animals, including birds [18,62,82], arboreal primates [59] and frogs [61], are characterized by rapid take-offs without the pronounced countermovement seen in more extended jumpers. Though reconstruction of posture in extinct animals presents many challenges of its own (e.g [4,24,36]), it is quite feasible that several documented postural transitions (for example, between avian and non-avian dinosaurs) could influence jumping through the outlined mechanisms. Therefore, we sought to assess the capacity of the human model to quantitatively and qualitatively capture the mechanical and performance differences observed when jumping from different starting postures. Humans represent an ideal case study in this respect since the control conditions (i.e. crouch to a specific depth) would be difficult if not impossible to elicit in non-human experimental subjects.

Simulated kinematic traces of *Model 1* generally align with the mechanical concepts outlined above for the respective starting postures. When jumping from full extension or a shallow crouch, the model performed a large countermovement, whereas a squat jump is used when starting from a deep crouch (figure 3). This pattern matches the differences in overall performance seen in the experimental data (figure 2*a*; table 4), and the observation that humans usually benefit from modest (several cm) gains to jump height when using countermovement mechanics [54,67]. These results also mirror the experimental kinematics (figure 3), though only in a broad sense, and some quantitative inconsistencies are apparent. In particular, the shallow crouch (and to a lesser extent the extended start) did not utilize a direct countermovement but instead underwent a period of limb extension prior to countermoving, which contrasted our experimental subject (figure 3*a,b,d,e*). While this initial extension phase serves to increase the eccentric (downwards) displacement distance, it does not appear to increase the concentric displacement distance. For example, the shallow crouch used a shorter concentric distance than the deep crouch (figure 2*b*), even though it employed a large countermovement (figure 3). During the time limit sensitivity analysis (electronic supplementary material, S2), we found that these preliminary joint extensions could be eliminated from the shallow crouch simulations by using shorter time intervals. However, this did result in a drop in performance, suggesting that the unusual kinematics of this solution (figure 3) have a temporal connection, which may explain why the shallow crouch still outperformed the deep crouch in terms of impulse (figure 2*e*) and overall performance (figure 2*a*). The time available to generate force is a critical factor in jumping [54,63,65,67,78,80], and the relatively short contact times used by *Model 1* simulations compared with the experiments (figure 2*d*) are likely to be one of the largest contributors to model underperformance, alongside reductions in PCSA.

There are several mechanical and physiological factors that will account for reductions in the contact time used by our simulations, and these primarily relate to artefacts arising from deliberate methodological simplifications to the modelling workflow. One particularly telling result is that this issue is much more pronounced in countermovement rather than squat jumping (figure 2*d*; table 4). While maximizing concentric distance is undoubtedly an important goal of countermovement jumping [18,64,66,68,80], there are other factors underlying this behaviour. It is well documented that skeletal muscle does not generate maximum force instantaneously *in vivo*, and that force must be built up over time, which is often termed an ‘active state’ [98]. Developing heightened levels of muscle activation is generally regarded as an additional purpose of a countermovement since this allows muscle activations to commence during the eccentric phase (i.e. during braking), improving force-generating capability during the concentric phase [54,55]. In contrast, our actuator model can generate the required level of force immediately, therefore circumventing the need to develop an active state. This simplification follows various studies on fossil animals (e.g. [7]), which use this approach because the physiological parameters needed to accurately replicate contractile dynamics are available from only a handful of modern taxa and difficult to generalize outside narrow phylogenetic and allometric margins. Though this method will undoubtedly produce unrealistically fast jumps, its effect on global force properties does not appear particularly severe (e.g. impulse, figure 2*e*; table 4), which likely reflects a tendency to replicate realistic patterns of force development (even if somewhat expedited and shifted towards higher forces over shorter intervals), that allow for timely co-ordination of joint movements (figure 3), and satisfy actuator force–length relationships. Therefore, while instantaneous muscle activations should have relatively minor effects on predicted jump height, they will tend to reduce the contact phase via elimination of eccentric-phase active state development (figure 2*d*; table 4). This means that in humans and other animals with habitually extended postures, which are likely to favour countermovement mechanics, our workflow will simulate disproportionately quick jumps compared with more crouched jumpers.

A second factor that contributes to shortened contact times is the use of a simplified, rigid foot, which encourages premature take-off where the aerial phase begins without reaching an equivalent level of limb extension to the experiments (figure 3). This will reduce the concentric distance and time available to generate force, meaning that take-off will occur with (i) the CoM at a lower position and (ii) travelling at a lower vertical velocity, which will necessarily equate to a reduction in jump height. In human jumpers, rapid rotation of the ankle and metatarsophalangeal joints late in the take-off sequence helps restrain motion of the proximal joints, allowing the powerful hip and knee extensors to perform more work overall since both hip and knee can be fully extended [51,63]. However, this mechanical solution is dependent upon a multi-segmented foot with toes capable of being rapidly rotated [51], whereas here the foot is modelled as a single fixed segment with immobile digits, which would limit its rotational ability and cause take-off to occur before the joints can be fully extended. Though splitting the foot into multiple mobile segments could theoretically solve this issue, it should be noted that foot mechanics are challenging to represent with reasonable biofidelity [31,32], and therefore a rigid functional foot is used in most simulations of bipedal animals [7,11,16,18–20,23–25,30–32,34].

To summarize, global performance of human *Model 1*, which incorporates subject-specific muscle mass information, showed good agreement with our experimental data, producing predictions that fall within the experimental range of measures and are −10 to −13% of mean values (figure 2; table 4). A general tendency towards underperformance by the simulations likely reflects some combination of reductions in extensor PCSA from fibre length estimation (around 20%, table 3), as well as reductions in the time available to generate force (figure 2*d*), due to premature take-off and disproportionately fast muscle activations. Joint kinematic traces demonstrated that the main strategic consideration of vertical jumping—do or do not use a countermovement—is correctly predicted and in line with the experiments (figure 3). However, simulations of shallow countermovements from intermediate starting postures do appear relatively challenging to optimize.

### 4.2. Accuracy of the avian model with real muscle masses

The jumping performance of our guineafowl model with real (measured) muscle masses (*Model 1*) was markedly lower than our equivalent human model, reaching just 56% of the maximum jump height predicted from Henry *et al*. [82] (figure 4*a*; table 5), despite having nearly double the extensor PCSA (table 3). These results are perhaps not unexpected given another recent predictive study of avian jumping also reported comparatively low simulated jump heights [18], and it is quite possible that important nuances of avian musculoskeletal morphology and physiology were lost or diluted in the simplified models used here. With these suspicions at hand, we performed several sensitivity analyses of muscle–tendon properties and starting posture, which showed that it is indeed possible for our model to achieve jump heights approaching those recorded experimentally (figure 4; table 5). However, these approaches rely on theoretical assumptions about avian biology that (ideally) require further investigation and furthermore may be difficult to generalize outside of crown group birds, amplifying the challenge of extending this procedure to evolutionary studies, such as assessments of non-avian theropods.

Before bird-specific factors are discussed, it is useful to first consider those issues already outlined by the human model. Subject-specific experimental performance data and aspects of model anatomy are a major strength of that model, but this is not so for the guineafowl, whose model can be considered a generalized representation of this taxon, with three-dimensional body segment geometries [71], muscle mass information [87] and experimental data [82], all hailing from separate sources with disparate methods and aims. While this is common practice in biomechanical modelling, there is potential for it to compromise model accuracy [15,41]. Indeed, these sources of uncertainty are wide-ranging and comprise: (i) estimation of the final jump height from the take-off position and velocity, (ii) use of a taxonomic average jump height instead of an individual average, (ii) estimation of starting joint angles with regards to the kinematic markers, (iv) using published limb muscle masses scaled to the size of our model and (v) PCSA comparisons made with optimized versus un-optimized fibre lengths (table 3). In addition, there are potentially also ‘unaccountable’ uncertainties, such as athletic disparities relating to differences in training and husbandry, which may also significantly impact overall performance in one or more of the groups of guineafowl we sourced data from. While none of these issues may be egregious by themselves, they could collectively introduce appreciable uncertainty to the validation process and thus deserve mention.

Another consideration is jumping strategy, which is loosely defined in avian studies, partly due to an (unavoidable) lack of experimental controls but also reflecting biological differences with humans. For one, it is important to note that most avian ‘jumping’ literature does not study jumping *sensu stricto*, but instead studies jumping as a component of flight, since the majority of the forces required for take-off (approx. 80–90%) are generated via the legs through a jumping manoeuvre [90,91,99]. Indeed, it is likely that jumping and take-off form a continuum in birds, where incorporating additional wingbeats to supplement the height of a submaximal jump is commonplace [82,100] and potentially economical [101]. Therefore, discussions of avian jumping on its own rest upon a handful of studies [18,62,82], where terminology is not always consistent, and the underlying mechanical principles are yet to be as rigorously codified.

Owing to their more crouched limb postures [96,102], the potential concentric distance habitually available to a bird is much greater than a human, and therefore most birds either squat jump, perform a short, fast countermovement or use some combination thereof [62,82,90,91,99,100,103]. Henry *et al*. [82] report a crouching behaviour in guineafowl experimentally but avoid describing this as a countermovement jump because of its relatively low speed (approx. 0.4s), and limited potential to increase storage of elastic energy. At face value, this would imply a squat jump, which would match the strategy used by *Model 1* (figure 5). However, we do not necessarily agree with this definition, given work on human jumping that suggests maximizing concentric distance is a viable goal of countermovements, especially at maximum effort and when speed is not a significant pressure [65,66,68,80]. Instead, we favour the definition used by Bishop *et al*. [18], where countermovements are viewed as a transient use of more flexed postures than the preceding or succeeding phases of the take-off sequence. This implies that guineafowl do use a form of countermovement, which helps explain the unweighting in their force data prior to limb extension [82] and places them in line with several other Galliformes, such as ptarmigans [62] and pheasants (SRRC 2023, unpublished data).

The discrepancy between the *Model 1* squat jump and experimental countermovements can explain some of the model’s underperformance. The starting posture used by *Model 1* approximates the initial flexion-extension presented by Henry *et al*. [82]. However, since those birds use a form of countermovement, they displace their CoM to a lower position by the start of the concentric phase thus increasing their total displacement distance relative to our model (figure 4*b*; table 5). We investigated this further using a more deeply flexed model and found that additional flexion can improve jump height by around 6 cm since the greater concentric distance allows larger impulses to be generated (figure 4*a*,*e*; table 5). However, this is not enough to meaningfully affect our overall interpretations regarding model accuracy, and its total impact on concentric distance is relatively small (2–3 cm; figure 4*b*; table 5). It is likely that the outstanding difference in concentric distance relates to some combination of premature take-off (the guineafowl model also includes a rigid functional foot, and the hip is not fully extended before take-off; figure 5) and size differences (our model is 1.38 kg compared with an experimental average body mass of 1.42 kg).

Why our original *Model 1* did not perform some form of countermovement is uncertain. Bishop *et al*. [18] simulated countermovement jumps in the Elegant crested tinamou (*Eudromia elegans*, another predominantly terrestrial bird) but used a much more extended starting posture and substantially longer contact periods, which would likely favour or encourage this movement pattern. In ptarmigans, the countermovement is of very low magnitude and quite fast (the entire take-off sequence is 0.28 s; [62]), which may be partially reflected in the guineafowl of Henry *et al*. [82] by an increase in the rate of knee flexion towards the end of the crouch phase. It is possible that the short, fast countermovements seen in birds (e.g. [62,90,91,99,100,103]) are strongly associated with eccentric phase force properties that have been overlooked in our models. Experimental and modelling studies on humans have demonstrated a trade-off between speed and height during vertical jumping [63–65,67], with shallow countermovements requiring shorter contact times [64,66–68] but emphasizing rapid eccentric force production that benefits muscle stretch-shortening and elastic recoil [54,64,80]. From an avian ecological standpoint, the crouched postures of birds mean that maximizing concentric distance is less important than humans, but jumping speed is probably critical (e.g. for predator evasion), thus short countermovements are both necessary given their morphology, and likely advantageous. However, the role of fast, shallow countermovements in generating high levels of eccentric force development may be (at least partly) circumvented when muscle activations are instantaneous, as they are in our models (discussed earlier in the human section). Under these conditions, squat jumping from a slightly higher position may produce equivalent jump heights while being kinematically easier to optimize.

Interestingly, the *Model 1* variants with longer muscle fibres (fibre length × 2; [22]) did perform a countermovement, which was reflected in their greater concentric distances (figure 4*b*; table 5) and overall jump heights (figure 4*a*; table 5). Muscle fibre (= fascicle) length is one of the most important variables in biomechanical systems since at the most basic or fundamental level, for a fixed muscle volume, the force produced by skeletal muscle is determined by its PCSA and maximum isometric stress, where PCSA is proportional to fibre length. While this study calculated fibre length as the length change of the actuator as the joints were exercised across their full RoM, which is an approach that can readily be applied to fossil skeletons and aggregated muscles [21], this method makes an implicit assumption that the actuator is relatively well tuned to generate force across its entire length change, which is unlikely to accurately represent *in vivo* fibre operating ranges [16,22,88]. Therefore, it is usually necessary to apply a corrective tuning factor, with a value of 2 being preferred based on sensitivity analyses in quadrupedal mammals [22]. It is not surprising that applying this tuning factor to our model induced significant changes in kinematic strategy and performance; however, it is notable that performance increased when fibres were lengthened (+12% jump height relative to the original *Model 1*; figure 4*a*) since doubling fibre length directly equates to halving PCSA. That said, comparisons of model contractile velocities showed that, on average, the limb extensors shortened at a slower, more optimal rate in this variant, which led to a relatively higher work output by the actuator contractile elements (electronic supplementary material, S3), that allowed an appreciable impulse to be built up over a longer interval (figure 4*e*; table 5). Though performance does exceed *Model 1*, it remains far below experimental values (−32%; figure 4*a*; table 5) and is fairly similar to the more deeply crouched variant that sought to isolate the impact of a countermovement (approx. 4 cm different in jump height; figure 4*a*; table 5). This similarity provides further evidence that the performance benefits of avian countermovements are unlikely to account for the full discrepancy between experimental and simulated jump heights, and in the fibre length × 2 model, it is evident that increases in work done by the contractile element are largely offset by reductions in the contribution of the elastic elements (particularly the series element; electronic supplementary material, S3).

While a tuning factor of 2 is recommended from mammalian data [22] and may be suited for certain avian muscle groups, to our knowledge, an appropriate (i.e. empirically grounded) tuning factor for avian muscles has yet to be derived. Therefore, we opted to investigate the impact of reduced fibre lengths by essentially inversing the previous model and dividing the original fibre lengths by 2 (a tuning factor of 0.5). There is some evidence to suggest that *in vivo* fibre operating ranges in birds may be relatively narrow compared with humans and other mammals [16,88,89], and previous work on avian grounded running has demonstrated that narrow fibre tuning may enhance force generation from crouched postures, by placing muscles at more optimal parts of their force–length curves in these postures [19]. Furthermore, shortening fibres will (in our workflow) lead to increases in tendon length, which may benefit the elastic storage and release [82] that was hampered by the wider tuning factor (fibre length × 2). Preliminary trials indicated that the 0.5 tuning factor could only be applied to the distal musculature, as more proximal applications resulted in violations of the femoral stress constraints. Given that maximum-effort jumping probably entails greater skeletal loads than other high-effort locomotor activities [74], this suggests that skeletal loading may be one potential tool for reducing uncertainties in muscle architecture (i.e. delimiting minimum fibre lengths) in extinct taxa [7]. Naturally, application of this tuning factor to the distal actuators will greatly increase the model’s ability to generate force through increased PCSAs. However, we found that despite this potential, maximum jump height was very similar to the original *Model 1* and underperformed the experimental measures by −45% (table 5). In direct contrast to the fibre length × 2 model, the 0.5 tuned variant contracted its extensor actuators at higher (less optimal) velocities, which led to reductions in contractile work, but relatively proportionate increases in elastic contribution (particularly in the parallel element; electronic supplementary material, S3). This result (in combination with those from the wider tuning factor) contradicts straightforward assumptions about the model’s force-generating capacity and indicates a high sensitivity of this model towards the tuning of fibre and tendon properties, which presumably require careful selection to ensure sufficient contributions from both the muscular and elastic components.

Directly translating (or comparing) intrinsic properties such as fibre lengths, tendon lengths and pennation angles from real muscles to our aggregated actuators is fairly challenging [19], and the suitability of doing so will vary massively across the actuators depending on the number and architectural disparity of their contributing muscles. Indeed, this is one reason why other authors opt to model larger suites of muscles, even though muscle pooling can yield fairly accurate results (e.g. cost of transport; [11,34] and maximum running speed; [20,34]), and importantly, requires fewer overall assumptions since fewer properties must be individually estimated. That said, the resulting performance error associated with anatomical misrepresentation from modelled/simplified muscle architecture will undoubtedly be more acute for certain muscles and functional groupings than others, and it is beneficial to consider the potential effects of localized error, even if one-to-one comparisons with real musculature are tenuous.

An advantage of optimization-based approaches is that this uncertainty can be investigated in an objective manner by allowing the simulator to optimize the parameters in question. We did this for uniarticular extensor tendon lengths of *Model 1* and the fibre length × 2 variant, which were allowed to vary by +/−10% of their original length (a modest arbitrary figure that allows exploration of the underlying processes). When this optimization protocol was applied to the original *Model 1*, it resulted in a pronounced increase in performance and fairly accurate jump heights compared with the experiments (−7%; figure 4*a*; table 5). Evaluation of its contractile dynamics showed that the optimized uniarticular extensor tendons had been universally shortened by the maximum permissible amount of −10%, and this had greatly increased (in an absolute and relative sense) the work done by the elastic elements, but at the cost of low muscular work at high contractile speeds (electronic supplementary material, S3), which can be further related to the short contact time (figure 4*d*; table 5). While previous studies have suggested that birds may utilize elastic recoil to increase joint power and raise jump height [82], this is thought to primarily occur through partial decoupling of joint movements and muscle shortening, which allows the extensors to operate more optimally over their force–length and force–velocity curves (i.e. modulation) [82,104]. It is likely, therefore, that the shorter tendons selected by our simulator represent a more direct amplification of joint power, using high contractile speeds to accentuate elastic outputs while diminishing absolute and relative work done by the muscular component (electronic supplementary material, S3).

Surprisingly however, when this optimization procedure was extended to the fibre length × 2 model, no evidence was found for either improved elastic amplification or modulation, and the performance increase was much more modest (−30% versus the experimental average), being very similar to the original fibre length × 2 model in jump height (+3%) and contractile dynamics (electronic supplementary material, S3). Presumably, the slower contractile speeds (also seen in the original fibre length × 2 model) limit the model’s potential to benefit from elastic processes, which may further explain why only the pedal plantarflexor tendon was substantially shortened, since the TMTP will undergo the fastest excursion (figure 5*d*). Given the apparent importance in contractile speeds to model work/power output (electronic supplementary material, S3), and ultimately contact time (figure 4*d*) and impulse generation (figure 4*e*), further consideration of *V*_max_ may be beneficial alongside any future analyses of fibre/tendon tuning. Birds can generate high forces relatively quickly because of their higher proportion of fast-twitch muscle fibres and greater maximum shortening velocities [85,105], and to account for this fact, the *V*_max_ of our guineafowl was set to 14 L s^−1^ (i.e. higher than the 8.4 L s^−1^ used in our human model) based on *in vivo* contractile information from two turkey distal limb muscles [85]. This value was applied to all actuators however, and it remains possible that 14 L s^−1^ may be too fast for certain muscle groups, particularly proximal muscles that undergo greater total displacements, and could therefore erroneously skew their force–velocity relationships. Bishop *et al*. [18] showed that doubling *V*_max_ (from 10 L s^−1^ to 20 L s^−1^) in simulations of tinamou vertical jumping could lead to an approximately 20% increase in predicted jump height, although this was heavily weighted towards the ankle extensors. While these values do appear relatively high when considering the measured shortening velocities reported in the mallard gastrocnemius lateralis during take-off, at 1.41 L s^−1^ for a terrestrial medium and 4.12 L s^−1^ for an aquatic medium [106], they may be acceptable for Galliformes given their aptitude for rapid, burst-like motion [107]. Lastly, rapid force generation may partially alleviate the temporal requirements of building up ‘active state’ and could further explain how some birds avoid or minimise countermoving while still achieving substantial jump distances [62,82,90,91,99,100,103].

Vertical jumping (and biomechanics more broadly) has been less extensively studied in birds than in humans, and it is apparent from our results that further consideration of various mechanical and physiological factors is needed before avian jumps can be reproduced with the same accuracy as human ones. In our *Model 1* guineafowl, which incorporates known muscle mass data, maximum jump height underperforms experimental values by approximately 44% (figure 4*a*), which is much greater than the 10–15% error in the human model (figure 2*a*). The simulated kinematic strategy was also found to be incorrect; the model having performed a squat jump whereas a short countermovement was utilized experimentally (figure 5; [82]). To a certain extent, this error may reflect a generally higher level of uncertainty during the modelling process than was involved in the construction of its human counterpart. However, our sensitivity analyses have gone some way to identify several areas for improvement. In particular, the correct tuning of estimated muscle fibre and tendon lengths appears to be critical to model performance, both in terms of its effects on actuator contractile dynamics and direct impact upon calculations of PCSA. A future investigation of these properties would certainly benefit from integrating new anatomical and physiological information, and it would be particularly useful to repeat the fibre length change analyses detailed in Sellers *et al*. [22] upon a sample of living avians, with emphasis upon how operational interactions between fibre and tendon lengths can be replicated with greater biofidelity in our modelling process.

### 4.3. Accuracy of models with estimated (fossil) muscle masses

Muscle mass is inarguably one of the most important parameters for high-intensity activities such as running or jumping, but it cannot be measured from fossil material and must be estimated when studying extinct taxa (e.g. [4,20,22,24,34,35,43]). Analytically, it may be expected that estimation methods that yield higher muscle masses and larger PCSAs should produce straightforward and proportional increases in jump height. However, we find that these relationships can be readily influenced by the proportional fidelity of the estimation as well as mechanical optima favouring particular coordination strategies. This means that a model’s final performance may not always reflect the outcome predicted by its relative increase in force-generating ability.

The total limb muscle mass of human *Model 2* is +46% greater than our experimental subject, with 7/9 actuators overestimated, including at least one extensor acting across each joint (electronic supplementary material, Dataset 1). In contrast, total muscle mass is only + 23% greater in *Model 3,* with fewer (5/9) actuators overestimated—though this is very skewed towards the distal muscles, whose summed mass is + 170% greater than our subject (electronic supplementary material, Dataset 1). For both models, muscle mass estimation is shown to yield increases in PCSA, with *Model 2* representing a +24% increase in extensor PCSA over our subject, while *Model 3* is marginally lower at +22% (table 3). In joint-specific terms, *Model 2* has more PCSA available for extension of every joint compared with our subject, with this being relatively skewed towards the distal joints in similar proportions to *Model 1* (table 3). Such a distribution is not the case for *Model 3*, however, as the relatively high total PCSA of this model is driven entirely by a large increase in ankle PCSA (+117% in ankle extension), and PCSA at the other joints is actually lower than our subject (table 3). Because the actuators working across the ankle are estimated with relatively short muscle fibres, large overestimates of muscle mass at this joint will necessarily equate to substantial increases in PCSA. These results for *Models 2* and *3* contrast *Model 1*, which had a similar total and proportional PCSAs to our subject (the differences reflecting fibre length estimation, since the muscle masses were subject-specific inputs) and may be expected to illicit considerable differences in simulated jumping performance.

*Model 2* achieves the greatest simulated human jump heights, and marginally (2–3%) outperforms the experimental averages during countermovement jumping (figure 2*a*; table 5). This model represents a bird–human intermediate, but given that birds, particularly terrestrial birds, have relatively more muscular limbs than humans [3] and can generate relatively larger hindlimb forces [82,91], the performance increase can be succinctly explained by the large increase in extensor PCSA (table 3). That said, the kinematics used by *Model 2* are generally less accurate than *Model 1*, particularly for countermovement jumping where there is (i) extreme ankle dorsiflexion, (ii) moderately excessive knee flexion and (iii) an unusual midpoint extension phase at the hip not seen in the human subject (figure 3). This result is undoubtedly facilitated by the extremely powerful actuators of this model, which can counteract the high external joint moments resulting from these unnatural postures, and probably also accounts for the large concentric distances (equivalent to the experiments and higher than *Model 1*; figure 2*b*; table 5). However, these kinematic strategies are not particularly well co-ordinated, since optimal co-ordination in vertical jumping requires a proximo-distal pattern of joint movement to maximize positive muscle work [51]. Therefore, the positive mechanical work done by the actuators is likely to be proportionally low relative to the large increases in PCSA, which may account for why the absolute increase in performance in *Model 2* is not as large compared with *Model 1* as would be expected from its force-generating potential alone. In addition, take-off also occurs prematurely in *Model 2* without reaching full joint extension (figure 3), and this would further function to overturn potential performance gains that would be predicted from the high impulse and take-off velocities (figure 2*c*,*e*; table 5). While kinematic aspects of *Model 2*’s performance are less accurate than *Model 1*, it is worth acknowledging that the qualitative alignment of each discrete parameter continues to replicate the experimental trials in terms of the relative jump heights achieved from different starting postures (figure 2; table 5). This indicates that schematic approaches to muscle estimation are not necessarily unviable for comparative biomechanical research that seeks to examine, for example, evolutionary changes in habitual limb posture.

The muscle masses used in *Model 3* reflect allometric relationships between osteological surface areas and limb muscle masses in hominoids. Humans differ substantially from non-human apes in their skeletal and soft-tissue anatomy and have greater lower limb muscle mass [108], so it is plausible that hominoid scaling equations may tend to under- or overestimate muscle masses when bone areas (particularly long bone shaft areas) are used as a proxy. This appears to be particularly pronounced in the distal muscle groups, which have substantially overestimated muscle masses that when combined with their short (estimated) fibre lengths yield very large ankle extensor PCSAs (table 3). However, proximal PCSAs are comparatively low, and the hip extensors are the weakest of the three models (table 3). Given the importance of hip extension to vertical jumping in humans, particularly countermovement jumping [54,55,109], it is unsurprising that *Model 3* underperforms the experimental trials and only marginally outperforms *Model 1* (despite its +23% greater limb muscle mass and +49% greater total PCSA). Interestingly however, joint kinematic traces do show that *Model 3* performs arguably the cleanest countermovement jump from an extended start (figure 3), even if it struggles to achieve sufficient hip and knee RoM from a shallow crouch. When squat jumping from a deep crouch, *Model 3* outperforms *Model 2* in terms of contact time and impulse generation (figure 2*d,e*; table 5), though this does not translate to greater take-off velocities or jump heights (figure 2*a*,*c*; table 5). Kinematic trajectories suggest that *Model 3* has chosen to sacrifice timely hip extension in favour of maximizing work done at the ankle (extreme initial dorsiflexion followed by rapid plantarflexion). High muscle forces would be needed to counteract the very high ankle moment this posture would incur, and paired with the relatively long contact time, may account for a high ‘impulse’ that does not necessarily reflect positive work done on the CoM as a whole. *Model 3* also takes off prematurely without achieving full joint extension (figure 3), which also accounts for why its appreciable impulses and velocities do not equate to jump heights within the range of the experiments. Unlike *Models 1* and *2*, which closely approximate the PCSA distribution of the actual subject, *Model 3* incorporates phylogenetic allometric information to yield a notably dissimilar distribution. Despite these differences however, the general qualitative arrangement of the starting postures is once-again maintained in the performance metrics (figure 2; table 5).

Similar to the human, guineafowl *Model 2* is found to overestimate total limb muscle mass, though the absolute effect is much lower (+19%) compared with measured values [87], even if a similar proportion of actuators (8/11) are overestimated (electronic supplementary material, Dataset 1). Total PCSA in *Model 2* exceeds *Model 1* and therefore is roughly three times as great as real guineafowl (table 3). The proportional distribution of extensor PCSA in *Model 2* is proximally a good match with *Model 1*, though the distal proportions are unusual in that there is a large increase in pedal plantar- and dorsiflexor PCSA. This predominantly reflects our allocation of 10% limb muscle mass to the TMTP and does not appear to relate to fibre length estimations (as in several human models) since in relative terms these are not particularly short (table 2; Supplementary Dataset 1). For guineafowl *Model 3*, PCSA was estimated directly using the equations of Cuff *et al*. [43], and thus comparisons of limb muscle mass cannot be made. PCSA estimates are, however, much lower than the other models, and only around 66% of that measured experimentally (table 3). *Model 3* has the lowest extensor PCSA acting across every joint with the exception of the TMTP, and the distribution of PCSA in this model is comparatively distally skewed (table 3).

While guineafowl *Model 2* has the highest total PCSA, it is only + 27% greater than *Model 1* (table 3). This is reflective of guineafowl being moderately muscular bipeds—more so than humans and smaller birds, but less so than large ratites—and therefore within the range of bird–human values that were available [3]. This relative increase in PCSA allowed *Model 2* to slightly outperform *Model 1* (+6%), though this still equates to a notable underperformance (−40%) relative to the experimental data. Given the similar distribution of extensor PCSA across the limbs of *Models 1* and *2*, similarities in many aspects of their jumping performance (figure 4) and use of the same kinematic strategy (a squat jump; figure 5) should be expected.

The attachment area approach used to estimate PCSA in guineafowl *Model 3* uses actual muscle origins areas, as opposed to gross surface areas used for the *Model 3* human [34,43]. For many muscles, attachment boundaries are not clearly demarked, and it is difficult to reliably estimate their extent without introducing subjectivity, which can introduce substantial error into downstream biomechanical analyses [37,38]. Cuff *et al*. [43] suggested that their approach was better suited for certain muscles more than others, and that it had a general tendency to underestimate total limb PCSA. Here, we find that *Model 3* has the lowest total PCSA of the three models and underestimates the measured values for guineafowl (table 3). The relatively diminished ability of this model to generate force therefore explains the reduction in jumping performance compared with the other models (figure 4*a*). It is likely that the unusual variation of squat jumping used by this model, where the hip and knee are re-extended halfway through push-off, can be explained by the particularly low estimates of PCSA for these joints, whereas the distal (particularly ankle) estimates are more reliable (table 3).

While the methods used to estimate muscle mass and PCSA can lead to appreciable changes to jump height in both taxa (figures 2*a* and 4a; table 5), they are perhaps less impactful as might be expected from absolute changes in PCSA (table 2 and 3). For example, there is a 35% decrease in total extensor PCSA between the highest (*Model 2*) and lowest (*Model 1*) human models jumping from full extension, but only a 13% decrease in jump height. Likewise, there is a 64% decrease in total extensor PCSA between the best (*Model 2*) and worst (*Model 3*) guineafowl models, yet this translates to only a 21% drop in jump height. These results suggest that simulations may be less sensitive to muscle estimation than would be intuitively expected, which we suspect may relate to coordination. Jumping strategies tend to be fairly conserved across models, and usually replicate a proximo-distal pattern of joint rotation (figures 3 and 4), which (as detailed earlier in the section) is considered optimal for maximizing muscular work and therefore vertical impulse and velocity [51]. Overestimation and proportional misrepresentation of all or some muscles would potentially overthrow these patterns. If the activation of certain muscles could no longer match those of others, this might lead to poor joint coordination, upset muscle force–length–velocity relationships, impact the spatial redistribution of work by the biarticular muscles [51] and lead to even more premature take-offs than already incurred. Previous work investigating the impact of muscle volume distribution during human squat jumping found that redistributing volume to muscles favourably configured for this movement (vastii, hamstrings and gastrocnemius) and away from those less favourably configured (rectus femoris and gluteus) could lead to jump heights around 15% greater than reference data, but with little change to kinematic strategy [110]. For these reasons, the optimal approach for most models may be a conservative tendency towards a narrow set of kinematic strategies, provided sufficient power is available to execute those specific movements. While novel kinematic strategies can arise from absolute and proportional differences in PCSA (e.g. human *Model 3* jumping from a deep crouch; figure 2), for the above reasons these may not be as well co-ordinated as a real experimental jumper or more accurate model, and performance may be lowered.

Though there is not necessarily a fixed number that defines an ‘acceptable’ or ‘good’ level of accuracy, that all human models upheld most of the qualitative performance differences observed experimentally across postures, while also being fairly close to the experiments in terms of absolute height, does indicate reasonably high predictive consistency. Likewise, many differences between human and guineafowl models, such as speed and relative jump height, are qualitatively consistent with their experimental equivalents, even with the lower absolute accuracy of the latter. The high levels of uncertainty in palaeobiological studies usually mean that relatively wider margins of error are acceptable, and qualitative rigor is just as (if not more) valuable than quantitative accuracy since this forms the basis of comparative discussion [3,4,7,18,22,24,34,35,37–39,43]. For example, while jump heights of our human models (*1–3*) range between −13% to within the values performed experimentally (figure 2), when viewed from a taxonomic perspective (i.e. ‘can we replicate *human* jump height’), which is the primary goal of nearly all evolutionary studies, then essentially all models are accurate since their performance is within the overall human range [63,64,66–68,78–80,94]. Of course, this does not extend to all parameters (e.g. joint kinematics, figure 3), which underscores the case-by-case reality of evaluating error in palaeobiological modelling, given the nature and magnitude of uncertainties involved [7,18,22,24,34,35,37–39,41,43,71].

## 5. Conclusions

In this study, we have evaluated the accuracy of forward dynamics simulations of maximum-effort vertical jumping in bipedal animals. The modelling procedure described herein and elsewhere [7,20–23,30–32,34] includes numerous techniques that expedite the simulation process (such as muscle and joint simplification) but which deviate substantially from reality and warrant assessment before being used to answer palaeobiological questions (with the additional uncertainties these entail). We show that when measured (i.e. known) muscle mass properties are used, we can achieve maximum jump heights within 10% of the experimentally measured range and between 10–13% of mean values recorded experimentally for a subject-specific human model jumping from three distinct starting postures. Conversely, the performance of an avian (guineafowl) model is worse, and this probably reflects higher degrees of anatomical, physiological and kinematic uncertainty during modelling, as well as the misrepresentation of several intrinsic muscle properties. A sensitivity analysis of the impact of muscle mass estimation—an unavoidable reality of modelling fossils—demonstrates that while this does impact overall jumping performance, the magnitude is lower than expected given the level of anatomical error, which may reflect the demands of joint and intermuscular co-ordination within conserved strategic optima. Exploring and explaining the (in)accuracy of an avian model is inherently more challenging than a human one because of lower experimental control, lack of a subject-specific dataset and a generally coarser understanding of avian biomechanics and locomotor physiology. That said, significant progress can likely be made with further experimental and modelling analyses of fibre and tendon lengths, which may be key to better understanding the exceptional *in vivo* performance of guineafowl and other birds. In a comparative sense, the current method may be most suitable when large differences in posture and morphology are at play and may be hypothesized to result in pronounced differences in jumping strategy. In such instances, qualitative differences in jump height and other parameters across different starting postures should be reliable.

## Data Availability

All numerical data used in this study are available in the extended supplementary material [111]. The original model input files, most optimal simulation output files, as well as the skeletal geometries are available at [112]. The source code for GaitSym 2017 can be found at https://github.com/wol101/GaitSym_2017.

## References

[1] Alexander RM. 1976 Estimates of speeds in dinosaurs. Nature **261**, 129–130.

[2] Rayfield EJ, Norman DB, Horner CC, Horner JR, Smith PM, Thomason JJ, Upchurch P. 2001 Cranial design and function in a large theropod dinosaur. Nature **409**, 1033–1037. (10.1038/35059070) 11234010

[3] Hutchinson JR. 2004 Biomechanical modeling and sensitivity analysis of bipedal running ability. I. extant taxa. J. Morphol. **262**, 421–440. (10.1002/jmor.10241) 15352201

[4] Hutchinson JR. 2004 Biomechanical modeling and sensitivity analysis of bipedal running ability. II. extinct taxa. J. Morphol. **262**, 441–462. (10.1002/jmor.10240) 15352202

[5] Pontzer H, Raichlen DA, Sockol MD. 2009 The metabolic cost of walking in humans, chimpanzees, and early hominins. J. Hum. Evol. **56**, 43–54. (10.1016/j.jhevol.2008.09.001) 18986682

[6] Soons J, Genbrugge A, Podos J, Adriaens D, Aerts P, Dirckx J, Herrel A. 2015 Is beak morphology in Darwin’s finches tuned to loading demands? PLoS One **10**, e0129479. (10.1371/journal.pone.0129479) 26068929 PMC4466803

[7] Sellers WI, Pond SB, Brassey CA, Manning PL, Bates KT. 2017 Investigating the running abilities of Tyrannosaurus rex using stress-constrained multibody dynamic analysis. PeerJ **5**, e3420. (10.7717/peerj.3420) 28740745 PMC5518979

[8] Cox PG, Rayfield EJ, Fagan MJ, Herrel A, Pataky TC, Jeffery N. 2012 Functional evolution of the feeding system in rodents. PLoS One **7**, e36299. (10.1371/journal.pone.0036299) 22558427 PMC3338682

[9] Liu G, Ren Y, Dong H, Akanyeti O, Liao JC, Lauder GV. 2017 Computational analysis of vortex dynamics and performance enhancement due to body–fin and fin–fin interactions in fish-like locomotion. J. Fluid Mech. **829**, 65–88. (10.1017/jfm.2017.533)

[10] De Groote F, Falisse A. 2021 Perspective on musculoskeletal modelling and predictive simulations of human movement to assess the neuromechanics of gait. Proc. R. Soc. B **288**, 20202432. (10.1098/rspb.2020.2432) PMC7935082 33653141

[11] Sellers WI, Dennis LA, Crompton RH. 2003 Predicting the metabolic costs of human bipedalism using evolutionary robotics. J. Exp. Biol. **203**, 1127–1136. (10.1242/jeb.00205) 12604572

[12] Nagano A, Komura T, Fukashiro S. 2007 Optimal coordination of maximal-effort horizontal and vertical jump motions – a computer simulation study. Biomed. Eng. OnLine **6**, 20. (10.1186/1475-925x-6-20) 17543118 PMC1896168

[13] Snively E, Cotton JR, Ridgely R, Witmer LM. 2013 Multibody dynamics model of head and neck function in Allosaurus (Dinosauria, Theropoda). Palaeontol. Electronic **16**, 1–29. (10.26879/338)

[14] Rankin JW, Rubenson J, Hutchinson JR. 2016 Inferring muscle functional roles of the ostrich pelvic limb during walking and running using computer optimization. J. R. Soc. Interface **13**, 20160035. (10.1098/rsif.2016.0035) 27146688 PMC4892259

[15] Charles JP, Grant B, D’Août K, Bates KT. 2020 Subject‐specific muscle properties from diffusion tensor imaging significantly improve the accuracy of musculoskeletal models. J. Anat. **237**, 941–959. (10.1111/joa.13261) 32598483 PMC7542200

[16] Bishop PJ, Krijn MB, Falisse A, Cuff AR, Allen VR, De Groote F, Hutchinson JR. 2021 Computational modelling of muscle fibre operating ranges in the hindlimb of a small ground bird (Eudromia elegans), with implications for modelling locomotion in extinct species. PLoS Comput. Biol. **17**, e1008843. (10.1371/journal.pcbi.1008843) 33793558 PMC8016346

[17] van Bijlert PA, Geijtenbeek T, Smit IH, Schulp AS, Bates KT. 2024 Muscle-driven predictive physics simulations of quadrupedal locomotion in the horse. Integr. Comp. Biol. **64**, 694–714. (10.1093/icb/icae095) 39003243 PMC11428545

[18] Bishop PJ, Falisse A, De Groote F, Hutchinson JR. 2021 Predictive simulations of musculoskeletal function and jumping performance in a generalized bird. Integr. Org. Biol. **3**, obab006. (10.1093/iob/obab006) 34377939 PMC8341896

[19] van Bijlert PA, van Soest AJ, Schulp AS, Bates KT. 2024 Muscle-controlled physics simulations of bird locomotion resolve the grounded running paradox. Sci. Adv. **10**, eado0936. (10.1126/sciadv.ado0936) 39321289 PMC11423892

[20] Sellers WI, Manning PL. 2007 Estimating dinosaur maximum running speeds using evolutionary robotics. Proc. R. Soc. B **274**, 2711–2716. (10.1098/rspb.2007.0846) PMC2279215 17711833

[21] Sellers WI, Manning PL, Lyson T, Stevens K, Margetts L. 2009 Virtual palaeontology: gait reconstruction of extinct vertebrates using high performance computing. Palaeontol. Electron **12**, 11A. https://palaeo-electronica.org/2009_3/180/index.html

[22] Sellers WI, Margetts L, Coria RA, Manning PL. 2013 March of the titans: the locomotor capabilities of sauropod dinosaurs. PLoS One **8**, e78733. (10.1371/journal.pone.0078733) 24348896 PMC3864407

[23] Bates KT, Manning PL, Margetts L, Sellers WI. 2010 Sensitivity analysis in evolutionary robotic simulations of bipedal dinosaur running. J. Vertebr. Paleontol. **30**, 458–466. (10.1080/02724630903409329)

[24] Bishop PJ, Cuff AR, Hutchinson JR. 2021 How to build a dinosaur: musculoskeletal modeling and simulation of locomotor biomechanics in extinct animals. Paleobiology **47**, 1–38. (10.1017/pab.2020.46)

[25] Bishop PJ, Falisse A, De Groote F, Hutchinson JR. 2021 Predictive simulations of running gait reveal a critical dynamic role for the tail in bipedal dinosaur locomotion. Sci. Adv. **7**, eabi7348. (10.1126/sciadv.abi7348) 34550734 PMC8457660

[26] van Bijlert PA, van Soest AJ’, Schulp AS. 2021 Natural Frequency Method: estimating the preferred walking speed of Tyrannosaurus rex based on tail natural frequency. R. Soc. Open Sci. **8**, 201441. (10.1098/rsos.201441) 33996115 PMC8059583

[27] Anderson L, Brassey C, Pond S, Bates K, Sellers WI. 2023 Investigating the quadrupedal abilities of Scutellosaurus lawleri and its implications for locomotor behavior evolution among dinosaurs. Anat. Rec. **306**, 2514–2536. (10.1002/ar.25189) 36896818

[28] Sellers WI, Margetts L, Bates KT, Chamberlain AT. 2013 Exploring diagonal gait using a forward dynamic three-dimensional chimpanzee simulation. Folia Primatol. **84**, 180–200. (10.1159/000351562) 23867835

[29] Charles JP, Cappellari O, Hutchinson JR. 2018 A dynamic simulation of musculoskeletal function in the mouse hindlimb during trotting locomotion. Front. Bioeng. Biotechnol. **6**, 61. (10.3389/fbioe.2018.00061) 29868576 PMC5964171

[30] Sellers WI, Dennis LA, Wang WJ, Crompton RH. 2004 Evaluating alternative gait strategies using evolutionary robotics. J. Anat. **204**, 343–351. (10.1111/j.0021-8782.2004.00294.x) 15198699 PMC1571306

[31] Sellers WI, Cain GM, Wang W, Crompton RH. 2005 Stride lengths, speed and energy costs in walking of Australopithecus afarensis : using evolutionary robotics to predict locomotion of early human ancestors. J. R. Soc. Interface **2**, 431–441. (10.1098/rsif.2005.0060) 16849203 PMC1618507

[32] Sellers WI, Pataky TC, Caravaggi P, Crompton RH. 2010 Evolutionary robotic approaches in primate gait analysis. Int. J. Primatol. **31**, 321–338. (10.1007/s10764-010-9396-4)

[33] Nagano A, Umberger BR, Marzke MW, Gerritsen KGM. 2005 Neuromusculoskeletal computer modeling and simulation of upright, straight‐legged, bipedal locomotion of Australopithecus afarensis (A.L. 288‐1). Am. J. Phys. Anthropol. **126**, 2–13. (10.1002/ajpa.10408) 15386246

[34] Bates KT, McCormack S, Donald E, Coatham S, Brassey CA, Charles J, O’Mahoney T, van Bijlert PA, Sellers WI. 2025 Running performance in Australopithecus afarensis. Curr. Biol. **35**, 224–230.(10.1016/j.cub.2024.11.025) 39701094

[35] Hutchinson JR, Garcia M. 2002 Tyrannosaurus was not a fast runner. Nature **415**, 1018–1021. (10.1038/416349b) 11875567

[36] Gatesy SM, Bäker M, Hutchinson JR. 2009 Constraint-based exclusion of limb poses for reconstructing theropod dinosaur locomotion. J. Vertebr. Paleontol. **29**, 535–544. (10.1671/039.029.0213)

[37] Bates KT, Wang L, Dempsey M, Broyde S, Fagan MJ, Cox PG. 2021 Back to the bones: do muscle area assessment techniques predict functional evolution across a macroevolutionary radiation? J. R. Soc. Interface **18**, 20210324. (10.1098/rsif.2021.0324) 34283941 PMC8292018

[38] Broyde S, Dempsey M, Wang L, Cox PG, Fagan M, Bates KT. 2021 Evolutionary biomechanics: hard tissues and soft evidence? Proc. R. Soc. B **288**, 20202809. (10.1098/rspb.2020.2809) PMC7935025 33593183

[39] Bates KT, Falkingham PL. 2018 The importance of muscle architecture in biomechanical reconstructions of extinct animals: a case study using Tyrannosaurus rex. J. Anat. **233**, 625–635. (10.1111/joa.12874) 30129185 PMC6183000

[40] Lieber RL, Fridén J. 2000 Functional and clinical significance of skeletal muscle architecture. Muscle Nerve **23**, 1647–1666. (10.1002/1097-4598(200011)23:113.3.co;2-d) 11054744

[41] Charles J, Kissane R, Hoehfurtner T, Bates KT. 2022 From fibre to function: are we accurately representing muscle architecture and performance? Biol. Rev. **97**, 1640–1676. (10.1111/brv.12856) 35388613 PMC9540431

[42] Bishop PJ, Wright MA, Pierce SE. 2021 Whole-limb scaling of muscle mass and force-generating capacity in amniotes. PeerJ **9**, e12574. (10.7717/peerj.12574) 34909284 PMC8638577

[43] Cuff AR, Wiseman ALA, Bishop PJ, Michel KB, Gaignet R, Hutchinson JR. 2023 Anatomically grounded estimation of hindlimb muscle sizes in Archosauria. J. Anat. **242**, 289–311. (10.1111/joa.13767) 36206401 PMC9877486

[44] Demuth OE, Wiseman ALA, van Beesel J, Mallison H, Hutchinson JR. 2022 Three-dimensional polygonal muscle modelling and line of action estimation in living and extinct taxa. Sci. Rep. **12**, 3358. (10.1038/s41598-022-07074-x) 35233027 PMC8888607

[45] Hutchinson B, Molnar K, Allen J, Makovicky V. 2011 A computational and comparative analysis of limb and body proportions in Tyrannosaurus rex with implications for locomotion and growth. PLoS ONE **6**, e26037. (10.1371/journal.pone.0097055) 22022500 PMC3192160

[46] Bates KT, Benson RBJ, Falkingham PL. 2012 A computational analysis of locomotor anatomy and body mass evolution in Allosauroidea (Dinosauria: Theropoda). Paleobiology **38**, 486–507. (10.1666/10004.1)

[47] Bates KT, Falkingham PL. 2012 Estimating maximum bite performance in Tyrannosaurus rex using multi-body dynamics. Biol. Lett. **8**, 660–664. (10.1098/rsbl.2012.0056) 22378742 PMC3391458

[48] Lautenschlager S. 2013 Cranial myology and bite force performance of Erlikosaurus andrewsi : a novel approach for digital muscle reconstructions. J. Anat. **222**, 260–272. (10.1111/joa.12000) 23061752 PMC3632231

[49] Pandy MG, Zajac FE, Sim E, Levine WS. 1990 An optimal control model for maximum-height human jumping. J. Biomech. **23**, 1185–1198. (10.1016/0021-9290(90)90376-e) 2292598

[50] Anderson FC, Pandy MG. 1999 A dynamic optimization solution for vertical jumping in three dimensions. Comput. Methods Biomech. Biomed. Eng. **2**, 201–231. (10.1080/10255849908907988) 11264828

[51] Bobbert MF, Knoek van Soest AJ. 2001 Why do people jump the way they do? Exerc. Sport Sci. Rev. **29**, 95–102. (10.1097/00003677-200107000-00002) 11474963

[52] Anderson FC, Pandy MG. 1993 Storage and utilization of elastic strain energy during jumping. J. Biomech. **26**, 1412–1427. (10.1016/0021-9290(93)90092-s) 8308046

[53] Bobbert MF. 2001 Dependence of human squat jump performance on the series elastic compliance of the triceps surae: a simulation study. J. Exp. Biol. **204**, 533–542. (10.1242/jeb.204.3.533) 11171304

[54] Bobbert MF, Gerritsen KGM, Litjens MCA, Van Soest AJ. 1996 Why is countermovement jump height greater than squat jump height? Med. Sci. Sports Exerc. **28**, 1402–1412. (10.1097/00005768-199611000-00009) 8933491

[55] Bobbert MF, Casius LJR. 2005 Is the effect of a countermovement on jump height due to active state development? Med. Sci. Sports Exerc. **208**, 440–446. (10.1249/01.mss.0000155389.34538.97) 15741843

[56] Nagano A, Komura T, Fukashiro S, Himeno R. 2005 Force, work and power output of lower limb muscles during human maximal-effort countermovement jumping. J. Electromyogr. Kinesiol. **15**, 367–376. (10.1016/j.jelekin.2004.12.006) 15811607

[57] Domire ZJ, Challis JH. 2007 The influence of squat depth on maximal vertical jump performance. J. Sports Sci. **25**, 193–200. (10.1080/02640410600630647) 17127594

[58] Emerson SB. 1985 Jumping and leaping. In Functional vertebrate morphology (eds M Hildebrand, DM Bramble, KF Liem), pp. 58–72. Cambridge, MA, USA: Harvard University Press.

[59] Crompton RH, Sellers WI, Günther MM. 1993 Energetic efficiency and ecology as selective factors in the saltatory adaptation of prosimian primates. Proc. R. Soc. B **254**, 41–45. 10.1098/rspb.1993.0124 8265674

[60] Toro E, Herrel A, Irschick D. 2004 The evolution of jumping performance in Caribbean Anolis lizards: solutions to biomechanical trade-offs. Am. Nat. **163**, 844–865. (10.1086/386347) 15266382

[61] Gomes FR, Rezende EL, Grizante MB, Navas CA. 2009 The evolution of jumping performance in anurans: morphological correlates and ecological implications. J. Evol. Biol. **22**, 1088–1097. (10.1111/j.1420-9101.2009.01718.x) 21462411

[62] Lees JJ, Folkow LP, Codd JR, Nudds RL. 2014 Seasonal differences in jump performance in the svalbard rock ptarmigan (Lagopus muta hyperborea). Biol. Open **3**, 233–239. (10.1242/bio.20147930) 24659246 PMC3988792

[63] Bobbert MF, Casius LJR, Sijpkens IWT, Jaspers RT. 2008 Humans adjust control to initial squat depth in vertical squat jumping. J. Appl. Physiol. **105**, 1428–1440. (10.1152/japplphysiol.90571.2008) 18719236

[64] Moran KA, Wallace ES. 2007 Eccentric loading and range of knee joint motion effects on performance enhancement in vertical jumping. Hum. Mov. Sci. **26**, 824–840. (10.1016/j.humov.2007.05.001) 17928080

[65] Domire ZJ, Challis JH. 2015 Maximum height and minimum time vertical jumping. J. Biomech. **48**, 2865–2870. (10.1016/j.jbiomech.2015.04.021) 25964210

[66] Salles AS, Baltzopoulos V, Rittweger J. 2011 Differential effects of countermovement magnitude and volitional effort on vertical jumping. Eur. J. Appl. Physiol. **111**, 441–448. (10.1007/s00421-010-1665-6) 20882293

[67] Jidovtseff B, Quievre J, Harris NK, Cronin JB. 2014 Influence of jumping strategy on kinematic and kinetic variables. J. Sports Med. Phys. Fit. **54**, 129–138. 24509983

[68] Sánchez-Sixto A, Harrison A, Floría P. 2018 Larger Countermovement Increases the Jump Height of Countermovement Jump. Sports **6**, 131. (10.3390/sports6040131) 30373113 PMC6316300

[69] Gatesy SM. 1990 Caudofemoral musculature and the evolution of theropod locomotion. Paleobiology **16**, 170–186. (10.1017/s0094837300009866)

[70] Hutchinson JR, Allen V. 2009 The evolutionary continuum of limb function from early theropods to birds. Naturwissenschaften **92**, 423–448. (10.1007/s00114-008-0488-3) 19107456

[71] Macaulay S, Hoehfurtner T, Cross SRR, Marek RD, Hutchinson JR, Schachner ER, Maher AE, Bates KT. 2023 Decoupling body shape and mass distribution in birds and their dinosaurian ancestors. Nat. Commun. **14**, 1575. (10.1038/s41467-023-37317-y) 36949094 PMC10033513

[72] Bramble DM, Lieberman DE. 2004 Endurance running and the evolution of Homo. Nature **432**, 345–352. (10.1038/nature03052) 15549097

[73] Crompton RH, Vereecke EE, Thorpe SKS. 2008 Locomotion and posture from the common hominoid ancestor to fully modern hominins, with special reference to the last common panin/hominin ancestor. J. Anat. **212**, 501–543. (10.1111/j.1469-7580.2008.00870.x) 18380868 PMC2409101

[74] Carrier DR, Schilling N, Anders C. 2015 Muscle activation during maximal effort tasks: evidence of the selective forces that shaped the musculoskeletal system of humans. Biol. Open **4**, 1635–1642. (10.1242/bio.014381) 26538637 PMC4736035

[75] Grant B, Charles J, Geraghty B, Gardiner J, D’Août K, Falkingham PL, Bates KT. 2022 Why does the metabolic cost of walking increase on compliant substrates? J. R. Soc. Interface **19**, 20220483. (10.1098/rsif.2022.0483) 36448287 PMC9709563

[76] Harman EA, Rosenstein MT, Frykman PN, Rosenstein RM. 1990 The effects of arms and countermovement on vertical jumping. Med. Sci. Sports Exerc. **22**, 825–833. (10.1249/00005768-199012000-00015) 2287261

[77] R Core Team. 2022 R: A language and environment for statistical computing. R foundation for statistical computing. Vienna, Austria. See https://www.R-project.org/.

[78] Linthorne NP. 2001 Analysis of standing vertical jumps using a force platform. Am. J. Phys. **69**, 1198–1204. (10.1119/1.1397460)

[79] Kirby TJ, McBride JM, Haines TL, Dayne AM. 2011 Relative net vertical impulse determines jumping performance. J. Appl. Biomech. **27**, 207–214. (10.1123/jab.27.3.207) 21844609

[80] Barker LA, Harry JR, Mercer JA. 2018 Relationships between countermovement jump ground reaction forces and jump height, reactive strength index, and jump time. J. Strength Cond. Res. **32**, 248–254. (10.1519/jsc.0000000000002160) 28746248

[81] Blender Development Team. 2020 Blender (Version 2.90.1). See https://www.blender.org.

[82] Henry HT, Ellerby DJ, Marsh RL. 2005 Performance of guinea fowl Numida meleagris during jumping requires storage and release of elastic energy. J. Exp. Biol. **208**, 3293–3302. (10.1242/jeb.01764) 16109891

[83] Alexander RM. 1974 The mechanics of jumping by a dog (Canis familiaris). J. Zool. **173**, 549–573. (10.1111/j.1469-7998.1974.tb04134.x)

[84] Hill AV. 1938 The heat of shortening and the dynamic constants of muscle. Proc. R. Soc. B **126**, 136–195.

[85] Nelson FE, Gabaldón AM, Roberts TJ. 2004 Force–velocity properties of two avian hindlimb muscles. Comp. Biochem. Physiol. Part **137**, 711–721. (10.1016/j.cbpb.2004.02.004) 15123179

[86] Rubenson J, Henry HT, Dimoulas PM, Marsh RL. 2006 The cost of running uphill: linking organismal and muscle energy use in guinea fowl (Numida meleagris). J. Exp. Biol. **209**, 2395–2408. (10.1242/jeb.02310) 16788023

[87] Cox SM, Easton KL, Lear MC, Marsh RL, Delp SL, Rubenson J. 2019 The interaction of compliance and activation on the force-length operating range and force generating capacity of skeletal muscle: a computational study using a guinea fowl musculoskeletal model. Integr. Org. Biol. **1**, obz022. (10.1093/iob/obz022) 32510037 PMC7259458

[88] Arnold EM, Delp SL. 2011 Fibre operating lengths of human lower limb muscles during walking. Phil. Trans. R. Soc. B **366**, 1530–1539. (10.1098/rstb.2010.0345) 21502124 PMC3130447

[89] Burkholder TJ, Lieber RL. 2001 Sarcomere length operating range of vertebrate muscles during movement. J. Exp. Biol. **204**, 1529–1536. (10.1242/jeb.204.9.1529) 11296141

[90] Heppner FH, Anderson JGT. 1985 Leg Thrust important in flight take-off in the pigeon. J. Exp. Biol. **114**, 285–288. (10.1242/jeb.114.1.285)

[91] Earls KD. 2000 Kinematics and mechanics of ground take-off in the starling Sturnis Vulgaris and the quail Coturnix Coturnix. J. Exp. Biol. **203**, 725–739. (10.1242/jeb.203.4.725) 10648214

[92] Bobbert MF, Huijing PA, van Ingen Schenau GJ. 1986 A model of the human triceps surae muscle-tendon complex applied to jumping. J. Biomech. **19**, 887–898. (10.1016/0021-9290(86)90184-3) 3793737

[93] Finni T, Komi PV, Lepola V. 2000 In vivo human triceps surae and quadriceps femoris muscle function in a squat jump and counter movement jump. Eur. J. Appl. Physiol. **83**, 416–426. (10.1007/s004210000289) 11138584

[94] Moir GL. 2008 Three different methods of calculating vertical jump height from force platform data in men and women. Meas. Phys. Educ. Exerc. Sci. **12**, 207–218. (10.1080/10913670802349766)

[95] Hutchinson JR, Anderson FC, Blemker SS, Delp SL. 2005 Analysis of hindlimb muscle moment arms in Tyrannosaurus rex using a three-dimensional musculoskeletal computer model: implications for stance, gait, and speed. Paleobiology **31**, 676–701. (10.1666/04044.1)

[96] Cross SRR, Marmol-Guijarro AC, Bates KT, Marrin JC, Tickle PG, Rose KA, Codd JR. 2024 Testing the form-function paradigm: body shape correlates with kinematics but not energetics in selectively-bred birds. Commun. Biol. **7**, 900. (10.1038/s42003-024-06592-w) 39048787 PMC11269648

[97] Crompton RH, Pataky TC, Savage R, D’Août K, Bennett MR, Day MH, Bates KT, Morse S, Sellers WI. 2012 Human-like external function of the foot, and fully upright gait, confirmed in the 3.66 million year old laetoli hominin footprints by topographic statistics, experimental footprint-formation and computer simulation. J. R. Soc. Interface **9**, 707–719. (10.1098/rsif.2011.0258) 21775326 PMC3284127

[98] Hill AV. 1950 the development of the active state of muscle during the latent period. Proc. R. Soc. B Biol. Sci. **137**, 320–329. 14786302 10.1098/rspb.1950.0043

[99] Bonser RHC, Rayner JMV. 1996 Measuring leg thrust forces in the common starling. J. Exp. Biol. **199**, 435–439. (10.1242/jeb.199.2.435) 9318079

[100] Tobalske BW, Altshuler DL, Powers DR. 2004 Take-off mechanics in hummingbirds (Trochilidae). J. Exp. Biol. **207**, 1345–1352. (10.1242/jeb.00889) 15010485

[101] Chin DD, Lentink D. 2017 How birds direct impulse to minimize the energetic cost of foraging flight. Sci. Adv. **3**, e1603041. (10.1126/sciadv.1603041) 28560342 PMC5435416

[102] Gatesy SM, Biewener AA. 1991 Bipedal locomotion: effects of speed, size and limb posture in birds and humans. J. Zool. **224**, 127–147. (10.1111/j.1469-7998.1991.tb04794.x)

[103] Provini P, Tobalske BW, Crandell KE, Abourachid A. 2012 Transition from leg to wing forces during take-off in birds. J. Exp. Biol. **215**, 4115–4124. (10.1242/jeb.074484) 22972887

[104] Roberts TJ, Marsh RL. 2003 Probing the limits to muscle-powered accelerations: lessons from jumping bullfrogs. J. Exp. Biol. **206**, 2567–2580. (10.1242/jeb.00452) 12819264

[105] Medler S. 2002 Comparative trends in shortening velocity and force production in skeletal muscles. Am. J. Physiol. Regul. Integr. Comp. Physiol. **283**, 368–378. (10.1152/ajpregu.00689.2001) 12121850

[106] Taylor-Burt KR, Biewener AA. 2020 Aquatic and terrestrial takeoffs require different hindlimb kinematics and muscle function in mallard ducks. J. Exp. Biol. **223**, b223743. (10.1242/jeb.223743) 32587070

[107] Tobalske BW, Dial KP. 2000 Effects of body size on take-off flight performance in the Phasianidae (Aves). J. Exp. Biol. **203**, 3319–3332. (10.1242/jeb.203.21.3319) 11023852

[108] Payne RC, Crompton RH, Isler K, Savage R, Vereecke EE, Günther MM, Thorpe SKS, D’Août K. 2006 Morphological analysis of the hindlimb in apes and humans. I. muscle architecture. J. Anat. **208**, 709–724. (10.1111/j.1469-7580.2005.00433.x-i1) 16761973 PMC2100225

[109] Vanrenterghem J, Lees A, Lenoir M, Aerts P, De Clercq D. 2004 Performing the vertical jump: Movement adaptations for submaximal jumping. Hum. Mov. Sci. **22**, 713–727. (10.1016/j.humov.2003.11.001) 15063050

[110] Wong JD, Bobbert MF, van Soest AJ, Gribble PL, Kistemaker DA. 2016 Optimizing the distribution of leg muscles for vertical jumping. PLoS One **11**, e0150019. (10.1371/journal.pone.0150019) 26919645 PMC4769356

[111] Cross SRR, Charles JP, Sellers WI, Codd JR, Bates KT. 2025 Supplementary Material from: Exploring the Accuracy of Palaeobiological Modelling Procedures in Forward-Dynamics Simulations of Maximum-Effort Vertical Jumping. Figshare. (10.6084/m9.figshare.c.7817027) PMC12092134 40400511

[112] Cross SRR, Charles J, Sellers WI, Codd J, Bates KT. 2024 Data from Cross et al. exploring the accuracy of palaeobiological modelling procedures in forward-dynamics simulations of maximum-effort vertical jumping. University of Liverpool Research Data Catalogue (10.17638/datacat.liverpool.ac.uk/2862) PMC12092134 40400511

